# Synthesis of Lasalocid-Based Bioconjugates and Evaluation of Their Anticancer Activity

**DOI:** 10.1021/acsomega.1c05434

**Published:** 2022-01-07

**Authors:** Michał Antoszczak, Dagmara Otto-Ślusarczyk, Marta Kordylas, Marta Struga, Adam Huczyński

**Affiliations:** † Department of Medical Chemistry, Faculty of Chemistry, Adam Mickiewicz University, Uniwersytetu Poznańskiego 8, 61-614 Poznań, Poland; ‡ Chair and Department of Biochemistry, Faculty of Medicine, Medical University of Warsaw, Banacha 1, 02-097 Warsaw, Poland

## Abstract

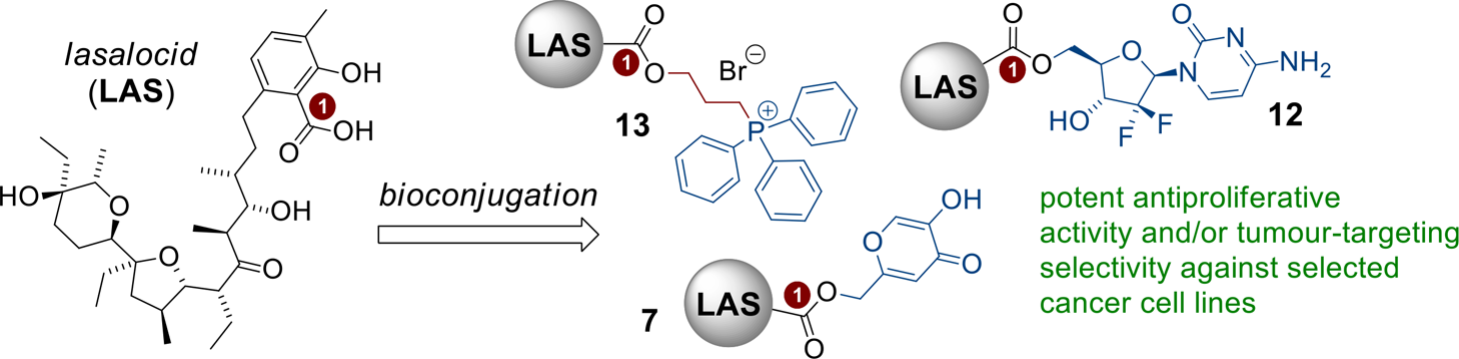

Using rationally designed bioconjugates is an attractive strategy to develop novel anticancer drugs with enhanced therapeutic potential and minimal side effects compared to the native structures. With respect to the promising activity of lasalocid (**LAS**) toward various cancer cells, this polyether ionophore seems to be an ideal candidate for bioconjugation. Herein, we describe the synthetic access to a cohort of nine conjugated products of **LAS**, in which the ionophore biomolecule was successfully combined via covalent bonds with selected anticancer therapeutics or other anticancer active components. The in vitro screening of a series of cancer cell lines allowed us to identify three products with improved anticancer activity profiles compared to those of the starting materials. The results indicate that human prostate cancer cells (PC3) and human primary colon cancer cells (SW480) were essentially more sensitive to exposure to **LAS** derivatives than human keratinocytes (HaCaT). Furthermore, the selected products were stronger inducers of late apoptosis and/or necrosis in PC3 and SW480 cancer cells, when compared to the metastatic variant of colon cancer cells (SW620). To establish the anticancer mechanism of **LAS**-based bioconjugates, the levels of interleukin 6 (IL-6) and reactive oxygen species (ROS) were measured; the tested compounds significantly reduced the release of IL-6, while the level of ROS was significantly higher in all the cell lines studied.

## 1 Introduction

The idea of bioconjugation, a chemical strategy used for the covalent derivatization of biomolecule(s), has been an intensively explored and rapidly progressing field of research,^1^ being a source of very intriguing hybrid structures. Indeed, the rationally designed bioconjugates have found application in chemical biology as probes for the investigation and visualization of biological systems and interactions, in biomaterial chemistry; but probably one of the most vital roles may be their potential utility as new therapeutic agents.^2−4^ The pivotal role of the bioconjugation chemistry is the development of efficient reactions with high selectivity and specificity that operate under mild conditions, appropriate for the biomolecules used.^5−7^

The group of attractive candidates to be conjugated with other bioactive components includes the naturally occurring polyether ionophores. These small molecules exhibit a very broad range of activities, such as their efficacy toward microorganisms and inhibitory effects on cancer cells.^8−11^ Prompted by the idea that tumor cells might be effectively destroyed by ionophores, conjugation of these compounds has emerged as an interesting direction of research. Nevertheless, despite the promising pharmacological profiles of natural ionophores, their pharmacophore hybridization seems to be a rather less explored avenue.

**Figure 1:**
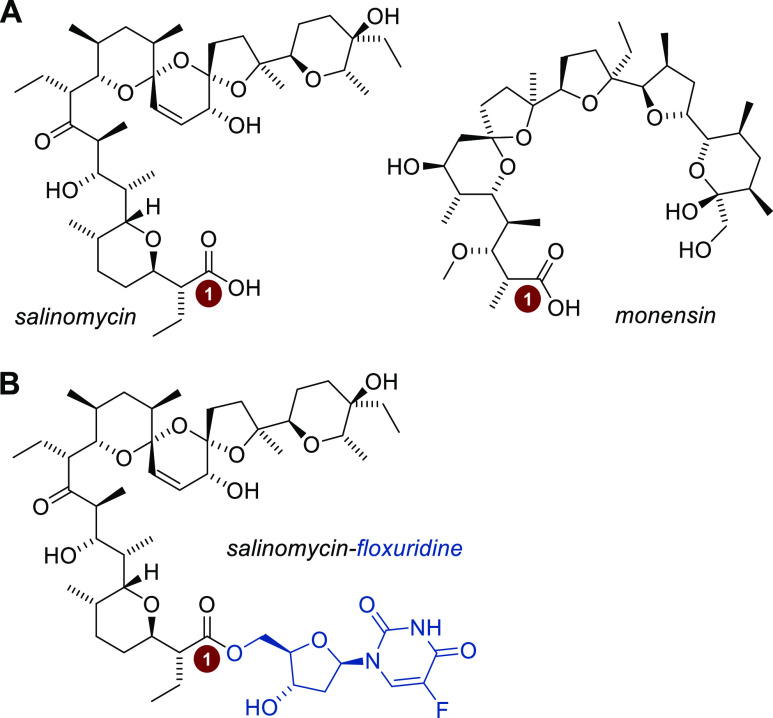
Structure of (A) monensin and salinomycin, two carboxyl polyether ionophores for which a bioconjugation strategy has been applied, and (B) salinomycin–floxuridine conjugated hybrid, a compound with very promising antiproliferative activity.

Till now, the application of the bioconjugation idea to develop novel anticancer agents has been limited mainly to two carboxyl ionophores, namely, monensin and salinomycin (Figure 1A).^12^ A combination of these biomolecules through covalent bonds has been successfully applied to amino acids, *Cinchona* alkaloids, flavonoids, or nucleosides, with most of the articles published in 2015.^13−19^ As far as the activity toward cancer cells is concerned, salinomycin conjugate with 5-fluoro-2′-deoxyuridine (floxuridine) (Figure 1B), a chemotherapeutic drug widely used toward selected human solid tumors,^20,21^ has shown even a few times more potent antiproliferative activity than that of the floxuridine precursor and two other reference anticancer drugs—cisplatin and doxorubicin.^17^

Besides monensin and salinomycin, also lasalocid (**LAS**, Scheme 2) has exhibited potent anticancer activity. In the tests toward various cancer cells, including breast, colon, as well as lung adenocarcinoma, this polyether ionophore produced by *Streptomyces lasaliensis* has shown higher antiproliferative activity, and simultaneously, lower cytotoxicity toward nontumor cells than those of cisplatin.^22^ In 2017, **LAS** was identified to induce cytotoxic apoptosis as well as cytoprotective autophagy via generating reactive oxygen species (ROS) in human prostate cancer PC3 cells.^23^ However, in the scientific literature, there is only limited information on the therapeutic effects of semisynthetic products derived from **LAS** on human cancer cells,^22^ and to the best of our knowledge, except two **LAS** dimeric structures with salinomycin reported recently,^24^ there is no information on the anticancer activity of other **LAS**-based bioconjugates.

In the past several years, various synthetic strategies have been developed and further used in bioconjugation chemistry, such as copper(I)-catalyzed alkyne-azide cycloaddition (CuAAC), strain-promoted azide-alkyne cycloaddition (SPAAC), and other bioorthogonal reactions,^25^ and also the hybridization of individual partners through rationally designed linkers. Another valuable synthetic strategy also seems to be the direct conjugation of the bioactive components without any previous manipulation.^26^ This methodology has been extensively used in the chemical modification of biomaterials (peptides, proteins, and polysaccharides) and the coupling of functional molecules in the biological field, but it requires the presence of compatible functional groups in the structure of modified compounds, like carboxyl, amine, thiol, and/or hydroxyl. As previously reported for salinomycin,^13−17^ such protocols are very promising in the bioconjugation of polyether ionophores.

Taking all these into account, in this paper, we describe the synthetic access to a series of nine bioconjugates of **LAS** with other biologically active components, conjugated together directly or by appropriate linkers (Scheme 2). As partners for hybridization with **LAS**, we selected compounds that exhibit activity against different types of tumor cells, like commonly used oncological drugs (5-fluorouracil, floxuridine, and gemcitabine),^27^ pentacyclic triterpenoid betulinic acid,^28^ kojic acid,^29^ as well as structural motifs widely used in medicinal chemistry to improve the biological activity profiles of targeted products, like ferrocene^30^ and triphenylphosphonium (TPP) moieties^31^ (Scheme 2). Our goal was to synthesize hybrids of **LAS** showing a wider therapeutic window when compared to the parent component(s), that is, analog structures with increased potential to kill cancer cells, and/or improved tumor-targeting selectivity. Therefore, all newly synthetized conjugates were evaluated in vitro for their cytotoxic activity using the MTT method. The biological activity studies included the cytotoxicity, proapoptotic activity, and interleukin-6 release assays that were performed on primary and metastatic cancer cell lines, as well as nontumor cells.

## 2 Results and Discussion

### 2.1 Design and Synthesis of Bioconjugates

**Scheme 1:**
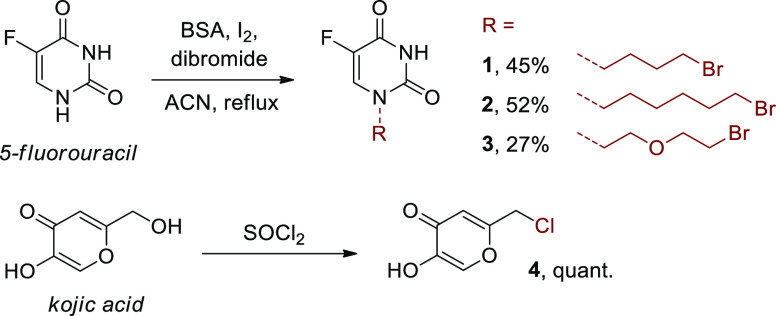
Synthesis of Precursors of 5-Fluorouracil and Kojic Acid

**Scheme 2:**
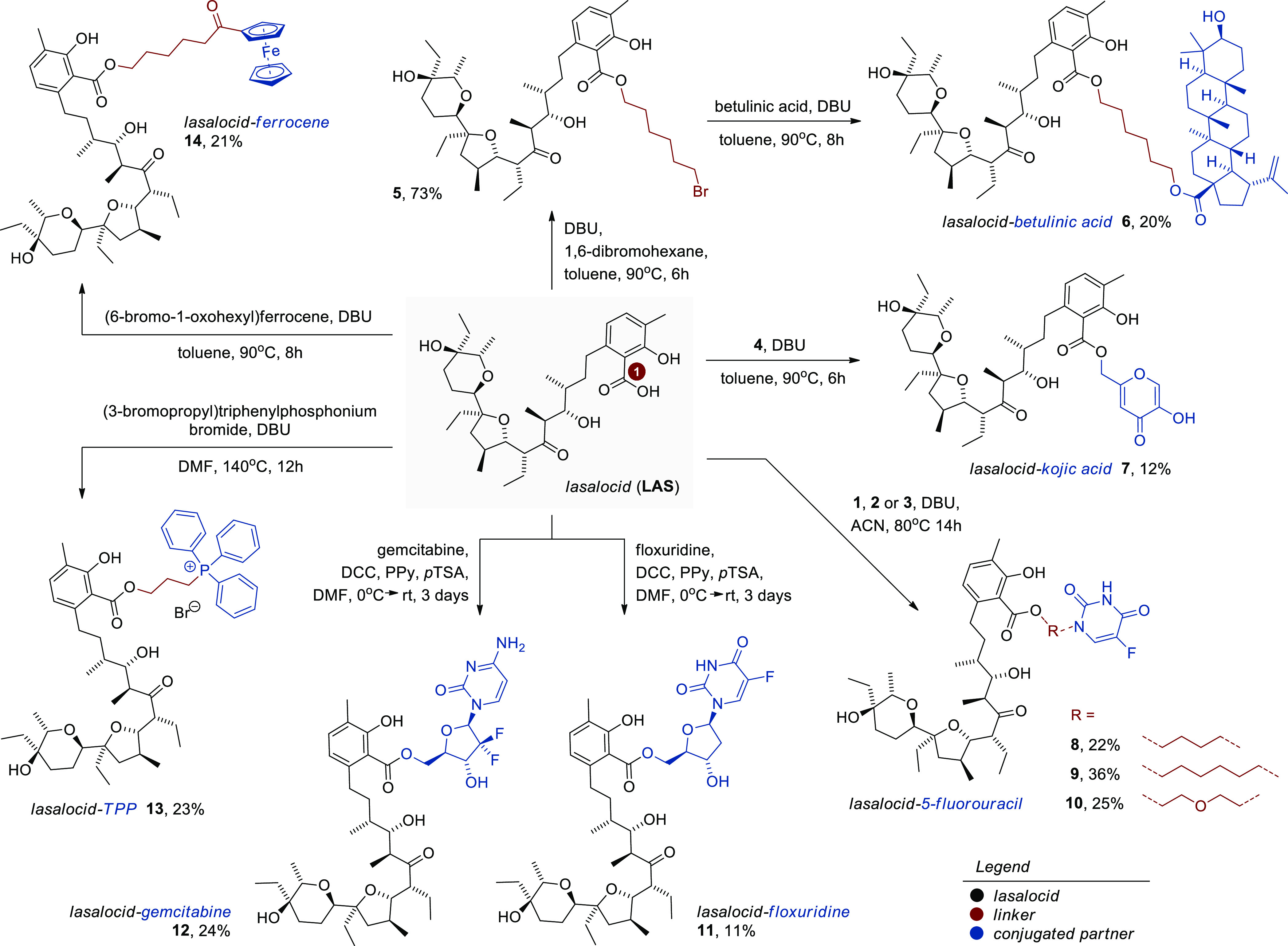
Synthesis of **LAS**-Based Bioconjugates

Motivated by the relatively high cytotoxic activity of bioconjugates of monensin and salinomycin combined at the C1 carboxyl via ester linkers with other bioactive components toward a panel of cancer cells,^13,16,17^ we decided to apply a similar methodology also to **LAS**. For this purpose, some of the partners dedicated for bioconjugation with the **LAS** molecule needed to be transformed before to the corresponding precursor structures that could be chemically compatible with the C1 carboxyl of the ionophore (please see Scheme 1, compounds **1–4**, and Scheme 2, compound **5**).

Firstly, using *N*,*O*-bis(trimethylsilyl)acetamide (BSA) and a catalytic amount of I_2_, 5-fluorouracil reacted with commercially available dibromides to form **1–3** with satisfactory yields (27–52%, Scheme 1). The linkers introduced into the 5-fluorouracil molecule differed not only in their length (butyl, hexyl), but also chemical nature (aliphatic, ether). Second, as the native structure of kojic acid did not show sufficient reactivity with the C1 carboxyl of **LAS**, it was necessary to convert the hydroxyl group of this molecule to a more reactive primary chloride (compound **4**, Scheme 1), which was quantitatively accomplished through the reaction of kojic acid with thionyl chloride.^32^

Finally, to conjugate **LAS** with betulinic acid, the ionophore was transformed in the DBU-promoted reaction with 1,6-dibromohexane to the corresponding ester analog **5** (73%, Scheme 2),^33^ fully compatible with the carboxyl group of betulinic acid. The NMR data of literature-known compounds **1**, **4**, and **5** were in good agreement with those found in the reference literature.^32−34^

Having access to the key precursors, in the next step, we synthesized a cohort of novel bioconjugates of **LAS** (compounds **6–14**, Scheme 2) with moderate yields; the synthetic procedures depended on the substrate used. If the starting material was an alcohol, it was the reaction with **LAS** in the presence of DCC, PPy (4-pyrrolidinopyridine), and catalytic pTSA (*para*-toluenesulfonic acid monohydrate) (Scheme 2). On the other hand, if primary chloride or bromide was applied, the conjugated hybrids were smoothly constructed by the S_N_2 esterification with the carboxyl group of **LAS** or betulinic acid, using DBU as a nucleophilic catalyst (Scheme 2).

The homogeneity and structure of the first time obtained bioconjugate products were determined using spectroscopic (FT-IR and NMR) and spectrometric (ESI MS) methods. The NMR spectra of newly synthesized compounds are given in Supporting Information (Figures S1–S30). Briefly, in the ^13^C NMR spectra of the **LAS** conjugates, the signal of the highest analytical significance was assigned to the newly introduced C1 ester group. Such a characteristic signal is observed in a very narrow range of 171.2–172.3 ppm, while the signal of the C1 carboxyl of chemically unmodified **LAS** is slightly shifted toward higher ppm values (173.2 ppm in chloroform-*d*^35^ and acetonitrile-*d*_3_,^36^ or 173.6 ppm in dichloromethane-*d*_2_^37^). In addition, both ^19^F NMR (for products **8–12**) and ^31^P NMR (for product **13**) spectra clearly confirmed the presence of the corresponding heteroatoms in the structure of the synthesized compounds.

### 2.2 Antiproliferative Activity

**LAS** and its newly synthesized bioconjugates **6–14**, together with their precursors, were evaluated for in vitro activity against three selected human cancer cell lines, that is, two colon cancer cell lines (SW480 and SW620) and one prostate cancer cell line PC3, using the MTT assay.^38^ Moreover, to evaluate the potential of the conjugated products for the selective targeting cancer cells, the human immortalized keratinocyte cell line HaCaT was also included in our studies.

The antiproliferative activities of the tested compounds were expressed as the values of the IC_50_ parameter (half maximal inhibitory concentration, i.e., the concentration of tested agents at which 50% inhibition is observed; shown in Table 1). Doxorubicin (DOX), a commonly used oncological drug, was used as a reference.

**Table 1:** Cytotoxicity (IC_50_, μM) and Tumor-Targeting Selectivity (SI) of the Studied Compounds Estimated by the MTT Assay^a^^,^^b^

	cancer cells						nontumor cells
compd	SW480		SW620		PC3		HaCaT
	IC_50_ (μM)	SI	IC_50_ (μM)	SI	IC_50_ (μM)	SI	IC_50_ (μM)
**LAS**	7.2 ± 0.79	2.2	6.1 ± 0.28	2.6	1.4 ± 0.05	11	16 ± 1.9
5FU	43 ± 3.1	1.2	39 ± 1.2	1.3	11 ± 0.97	4.7	52 ± 1.6
BET	105 ± 7.0	1.0	65 ± 3.7	1.7	71 ± 5.9	1.5	108 ± 1.3
FER	61 ± 0.98	1.9	55 ± 0.70	2.1	59 ± 0.78	1.9	115 ± 0.95
FLO	16 ± 1.2	2.4	8.8 ± 0.69	4.3	14 ± 0.57	2.7	38 ± 0.72
GEM	1.2 ± 0.05	1.3	3.4 ± 0.65	0.4	0.14 ± 0.02	11	1.5 ± 0.05
KOJ	119 ± 2.8	1.9	94 ± 4.0	2.4	56 ± 3.3	4.0	225 ± 7.1
TPP	46 ± 1.1	0.6	39 ± 2.5	0.7	31 ± 0.98	0.8	26 ± 0.96
**1**	36 ± 0.79	5.0	75 ± 2.7	2.4	308 ± 5.2	0.6	179 ± 4.3
**2**	103 ± 3.4	2.2	36 ± 0.83	6.2	3.1 ± 0.15	73	224 ± 2.8
**3**	55 ± 3.5	1.9	47 ± 3.8	2.2	42 ± 2.3	2.5	104 ± 2.2
**4**	28 ± 1.8	1.4	20 ± 2.6	2.0	20 ± 1.5	2.0	39 ± 0.45
**5**	42 ± 1.6	1.2	34 ± 1.4	1.5	18 ± 0.98	2.8	50 ± 0.34
**6**	320 ± 8.8	0.05	40 ± 2.1	0.4	200 ± 1.8	0.1	15 ± 0.59
**7**	7.8 ± 0.09	0.8	9.9 ± 0.08	0.6	3.8 ± 0.07	1.7	6.3 ± 0.10
**8**	67 ± 1.2	0.5	14 ± 1.0	2.4	3.6 ± 0.08	9.2	33 ± 3.4
**9**	66 ± 2.5	0.7	55 ± 1.5	0.8	36 ± 0.79	1.2	44 ± 1.8
**10**	22 ± 2.2	0.5	11 ± 1.4	1.1	12 ± 0.13	1.0	12 ± 0.59
**11**	31 ± 1.2	1.6	19 ± 2.0	2.6	13 ± 1.9	3.8	50 ± 0.59
**12**	12 ± 0.86	1.2	3.6 ± 1.1	3.9	1.6 ± 1.2	8.8	14 ± 0.22
**13**	1.4 ± 1.5	2.3	2.3 ± 2.2	1.4	1.8 ± 0.09	1.8	3.2 ± 0.12
**14**	76 ± 2.6	1.2	63 ± 2.9	1.5	3.3 ± 0.8	28	93 ± 3.9
DOX^c^	0.75 ± 0.10	0.4	0.26 ± 0.10	1.1	0.59 ± 0.02	0.5	0.29 ± 0.10

a 5FU = 5-fluorouracil, BET = betulinic acid, DOX = doxorubicin, FER = (6-bromo-1-oxohexyl)ferrocene, FLO = floxuridine, GEM = gemcitabine, KOJ = kojic acid, **LAS** = lasalocid, and TPP = (3-bromopropyl)triphenylphosphonium bromide.

b Data are expressed as mean ± SD; IC_50_ (μM), the concentration of the compound that corresponds to a 50% growth inhibition of the cell line (compared to the control) after culturing the cells for 72 h with the individual compound; SI (selectivity index) was calculated using the formula: SI = IC_50_ for normal cell line (HaCaT)/IC_50_ for respective cancer cell lines (SW480, SW620, or PC3); SW480, human primary colon cancer cell line; SW620, human metastatic colon cancer cell line; PC3, human prostate cancer cell line; HaCaT, human immortalized keratinocyte cell line.

c The selected reference compound commonly used in cancer treatment.

First of all, **LAS** and gemcitabine (GEM) were identified as the most promising antiproliferative agents among all the precursors tested; both compounds showed potent activity against three cancer cell lines at a relatively low micromolar concentration range (IC_50_ = 1.4–7.2 μM for **LAS** and IC_50_ = 0.14–3.4 μM for GEM). However, the polyether ionophore was found to target the tumor cells more selectively, as clearly indicated by the values of selectivity index (SI = 2.2–11 for **LAS** and SI = 0.4–11 for GEM).

The SI is an important pharmaceutical parameter. Briefly, SI > 1.0 identifies compounds with more potent activity against cancer cells than their toxicity toward nontumor cells.^39^ Using such criteria, of note is that a reference anticancer drug DOX inhibited the proliferation of cancer cells rather non-selectively, with SI = 0.4–1.1. Importantly, **LAS**–gemcitabine-conjugated product (compound **12**) merged the anticancer potential of both starting precursors, particularly for the human metastatic colon cancer cell line SW620. With improved antiproliferative activity compared to **LAS** (IC_50_ = 3.6 μM vs 6.1 μM) and more promising tumor-targeting selectivity than those of both native structures **LAS** and GEM (SI = 3.9 vs 0.4–2.6, respectively), bioconjugate **12** seems to be a potentially good candidate in the fight against this type of cancer.

It is an important finding, as both the survival outcome and treatment response of metastatic colon cancer patients are far from satisfactory. Metastases are the main cause of colon cancer-related mortality; it is estimated that ∼22% of colon cancers are metastatic at initial diagnosis, and even 70% of individuals may develop metastatic relapses.^40−42^ Furthermore, metastatic colon cancer patients face a rather poor prognosis, with a relative 5-year survival rate of only 14%.^43^ On the other hand, with respect to prostate cancer cell line PC3, compound **12** also showed potent antiproliferative activity (IC_50_ = 1.6 μM), together with good selectivity of action (SI = 8.8), but both these parameters were slightly less favorable when compared to parent **LAS** and GEM.

The group of semisynthetic products with improved biological activity profiles also comprises two other compounds, that is, **LAS**–TPP conjugate (compound **13**) and **LAS**–ferrocene conjugate (compound **14**). For compound **13**, the most promising results were obtained in the tests on the SW480 cancer cell line; the antiproliferative activity of the conjugated product was about five times more potent than that of **LAS** (IC_50_ = 1.4 μM vs 7.2 μM), with comparable values of the SI parameter (SI = 2.3 vs 2.2). Additionally, for two other cancer cell lines, compound **13** was found to inhibit proliferation at low micromolar concentrations, with the IC_50_ value against SW620 almost three times lower than that of the unmodified polyether ionophore (IC_50_ = 2.3 μM vs 6.1 μM), but with slightly less favorable tumor selectivity in this case. The antiproliferative activity of compound **14** seems to be strongly dependent on the variant of cancer cell line used. While the conjugated product selectively targeted the prostate cancer cell line PC3 (IC_50_ = 3.3 μM and SI = 28), it was almost completely ineffective toward both colon cancer cell lines.

Finally, the activities of **LAS**–kojic acid conjugate (compound **7**) and the conjugate of **LAS** and 5-fluorouracil hybridized together via the *n*-butyl linker (compound **8**) seem to be also worth noting. However, although both conjugated products displayed potent antiproliferative activity against PC3 cancer cell line, their IC_50_ (3.6–3.8 μM) and SI values (1.7–9.2) were less promising than those of the starting **LAS**. The antiproliferative activity of **LAS**-based bioconjugates could be a consequence of the cleavage of ester bonds by cellular esterases,^44^ which finally leads to the release of the highly cytotoxic precursors (promoieties); a similar trend has been observed previously for salinomycin conjugates with floxuridine.^17^

### 2.3 Apoptotic Activity

**Figure 2:**
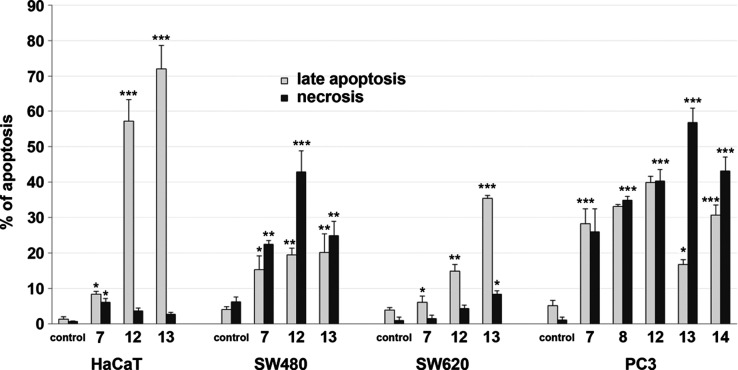
Effects of **LAS**-based bioconjugates **7**, **8**, **12**, **13**, and **14** on late apoptosis or necrosis in HaCaT, SW480, SW620, and PC3 cells. The cells were incubated for 72 h with the respective compounds at their IC_50_ concentrations, then the cells were harvested, stained with Annexin V-FITC and PI, and analyzed using flow cytometry. Data are expressed as % of cells at the late stage of apoptosis or necrosis and as means ± SD. ***p ≤ 0.001, **p ≤ 0.01, *p ≤ 0.05, as compared to the control.

**Figure 3:**
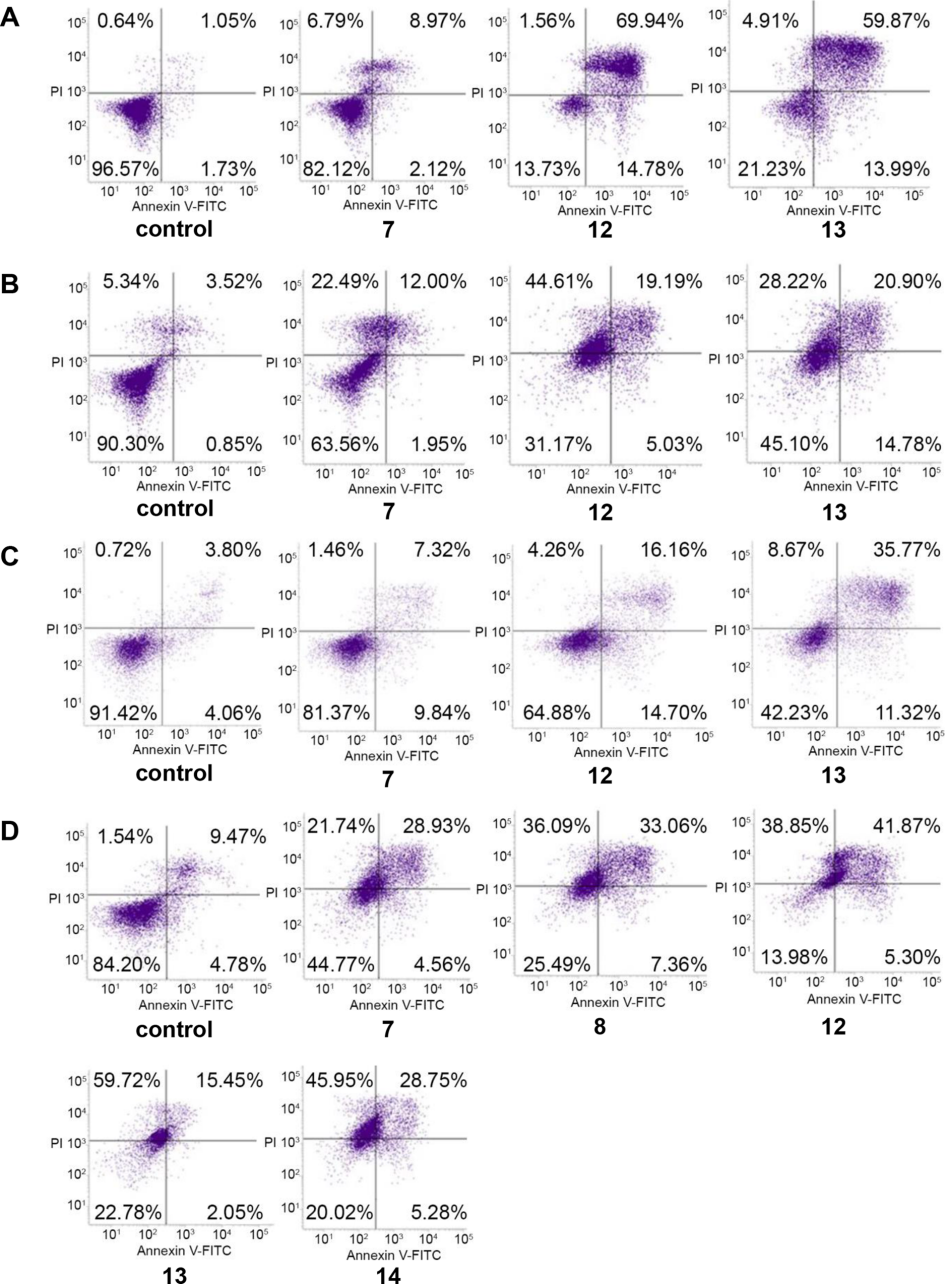
Effects of **LAS**-based bioconjugates **7**, **8**, **12**, **13**, and **14** on early/late apoptosis or necrosis in (**A**) HaCaT, (**B**) SW480, (**C**) SW620, and (**D**) PC3 cells detected with Annexin V-FITC/PI by flow cytometry. Diagrams show results of representative experiments. The lower right quadrant shows early apoptotic cells (Annexin V-FITC positive and PI negative staining); the upper right and upper left quadrants represent the late stage of apoptotic or necrotic cells (Annexin V-FITC-positive and PI-positive or Annexin V-FITC-negative and PI-positive staining, respectively).

To gain more information on the in vitro mechanism of action of **LAS** derivatives, the effects of the most promising bioconjugates **7**, **12**, and **13** on early and late apoptosis or necrosis were estimated using flow cytometry analysis (Figures 2 and 3); compounds **8** and **14** were also included, as they exhibited antiproliferative activity toward prostate cancer cells at low micromolar concentrations (IC_50_ = 3.6 μM and IC_50_ = 3.3 μM, Table 1).

The incubation of SW480, SW620, and PC3 cancer cells with **LAS**-based bioconjugates indicated the significantly higher percentage of those in late apoptosis or necrosis when compared to the control (untreated cells). In SW620 cells, the strongest late apoptosis-inducing effect was detected for compound **13**, while in the corresponding SW480 cells, two analog structures (compounds **12** and **13**) were identified as particularly promising in this regard (Figure 2).

However, it should be noted that compound **13** gave a similar percentage of late apoptotic as well as necrotic SW480 cells (20.15 and 24.82%, respectively), while compounds **7** (28.24 and 26.00%), **8** (33.16 and 34.88%), and **12** (39.92 and 40.31%) demonstrated a similar trend in the PC3 cancer cell line (Figures 2 and 3). Compound **12** induced early and late apoptosis at a similar level also in SW620 cells (14.53 and 14.79%, respectively) (Figure 3).

The strongest necrotic activity was found for compounds **13** (56.81%) and **14** (43.12%) in PC3 cells, and compounds **7** and **12** (22.39 and 42.85%, respectively) in SW480 cells (Figures 2 and 3). The treatment of HaCaT cells with compounds **12** and **13** also revealed a high level of late apoptosis or necrosis (Figures 2 and 3); both compounds were the strongest activators of late apoptosis, which varied from 57.26 to 72.01%. The results obtained from the apoptosis assay were consistent with those obtained by the MTT method for all the studied cancer cell lines.

### 2.4 Interleukin-6 Assay

**Figure 4:**
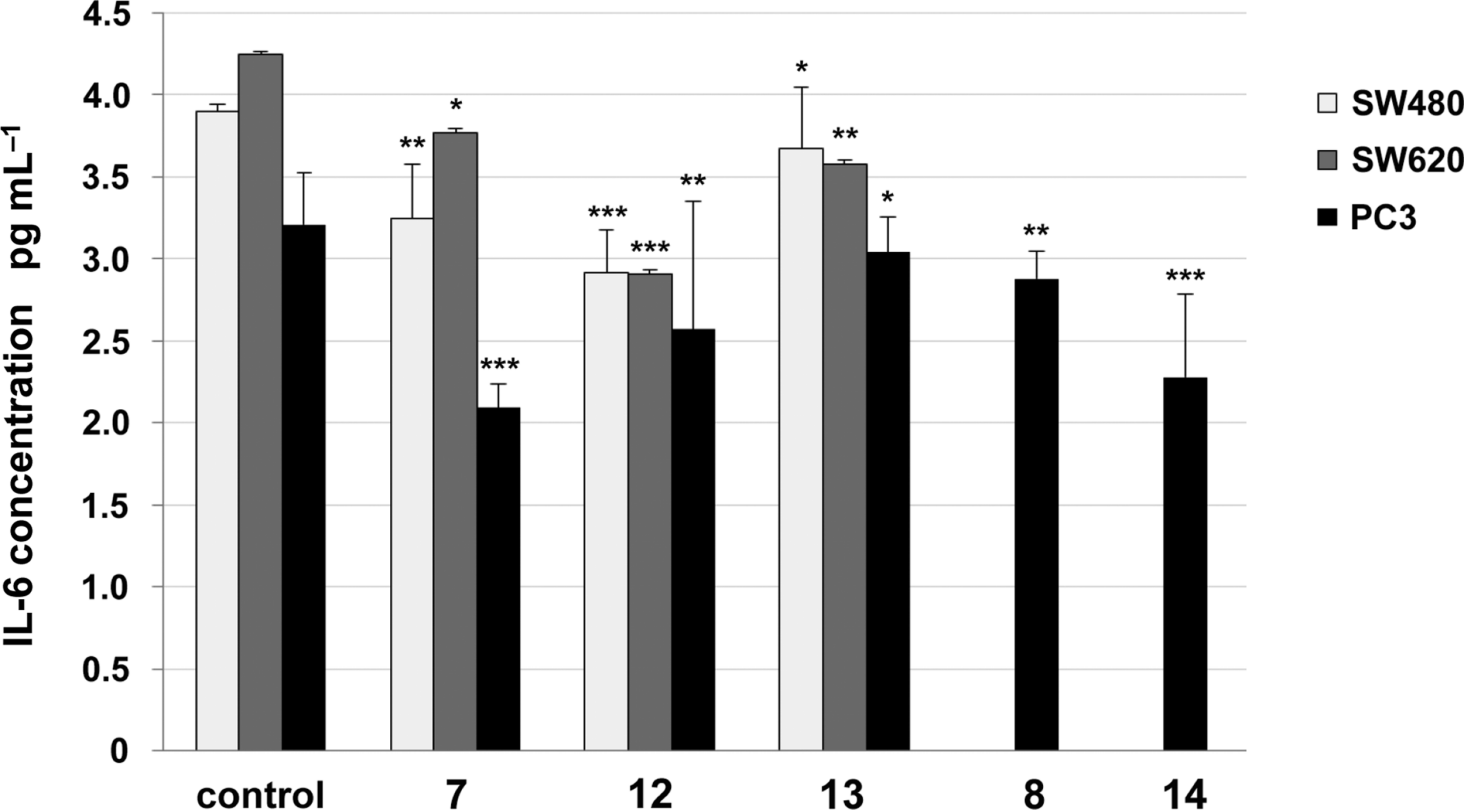
Effects of **LAS**-based bioconjugates **7**, **8**, **12**, **13**, and **14** on IL-6 levels. The levels of IL-6 in culture supernatant were measured by the ELISA test. Data are expressed as the mean ± SD from three independent experiments performed in triplicate. ***p ≤ 0.001, **p ≤ 0.01, *p ≤ 0.05, as compared to the control.

To extend the study on the anticancer mechanism of action of **LAS** bioconjugates, the interleukin 6 (IL-6) assay was performed (Figure 4). IL-6 is a pleiotropic proinflammatory cytokine.^45,46^ As colon and prostate cancers are associated with inflammation, IL-6-based mechanisms may be involved in tumor development; its increased expression has been related to the advanced stages of the disease as well as decreased survival in colorectal cancer patients.^47^ The serum IL-6 levels have been also correlated with prostate tumor burden and patient morbidity.^48^ IL-6 has exerted oncogenic effects in various inflammation-associated cancers via activation of multiple signaling pathways, including Janus kinases (JAKs) and signal transducers and activators of transcription 3 (STAT3),^47^ promoting tumor initiation and growth in both colon and prostate cancer.^49,50^ Of note, IL-6 has been found to be able to convert poorly differentiated colon and prostate cancer cells into cancer stem cells that are resistant to conventional radio- and chemotherapy.^51,52^ However, to the best of our knowledge, the changes in the IL-6 levels induced by semisynthetic derivatives of **LAS** in colon and prostate cancer cells have not been studied until now.

All three human cancer cell lines were treated with the IC_50_ concentrations of the most promising **LAS** bioconjugates, that is, compounds **7**, **8**, **12**, **13**, and **14** (Figure 4). In the presence of **LAS** derivatives, a significant reduction of IL-6 concentration was observed in the studied cancer cell lines, with PC3 cells identified as the most sensitive to the inhibition of IL-6 release. Looking closer at the results, the strongest effects were observed for the structures of the two analogs (compounds **7** and **14**), which inhibited IL-6 release in PC3 cells ∼1.2 and 1.0-fold more effectively, respectively, when compared to control (*p* ≤ 0.001). On the other hand, the treatment with compound **12** was more effective for both primary and metastatic colon cancer cell lines; this compound decreased IL-6 concentration ∼1.0 time for SW480 cells and 1.3 time for SW620 cells compared to the control.

### 2.5 ROS Production

Many studies have shown that regulation of the ROS level in various cancer cell types is a key strategy of anticancer drugs to induce apoptosis.^53,54^ Therefore, we examined the ability of two most promising **LAS** bioconjugates **12** and **13** at their IC_50_ concentrations (Table 1) to induce ROS production in cancer cell lines and normal HaCaT cells after 1, 4, 12, and 24 h of treatment.

**Figure 5:**
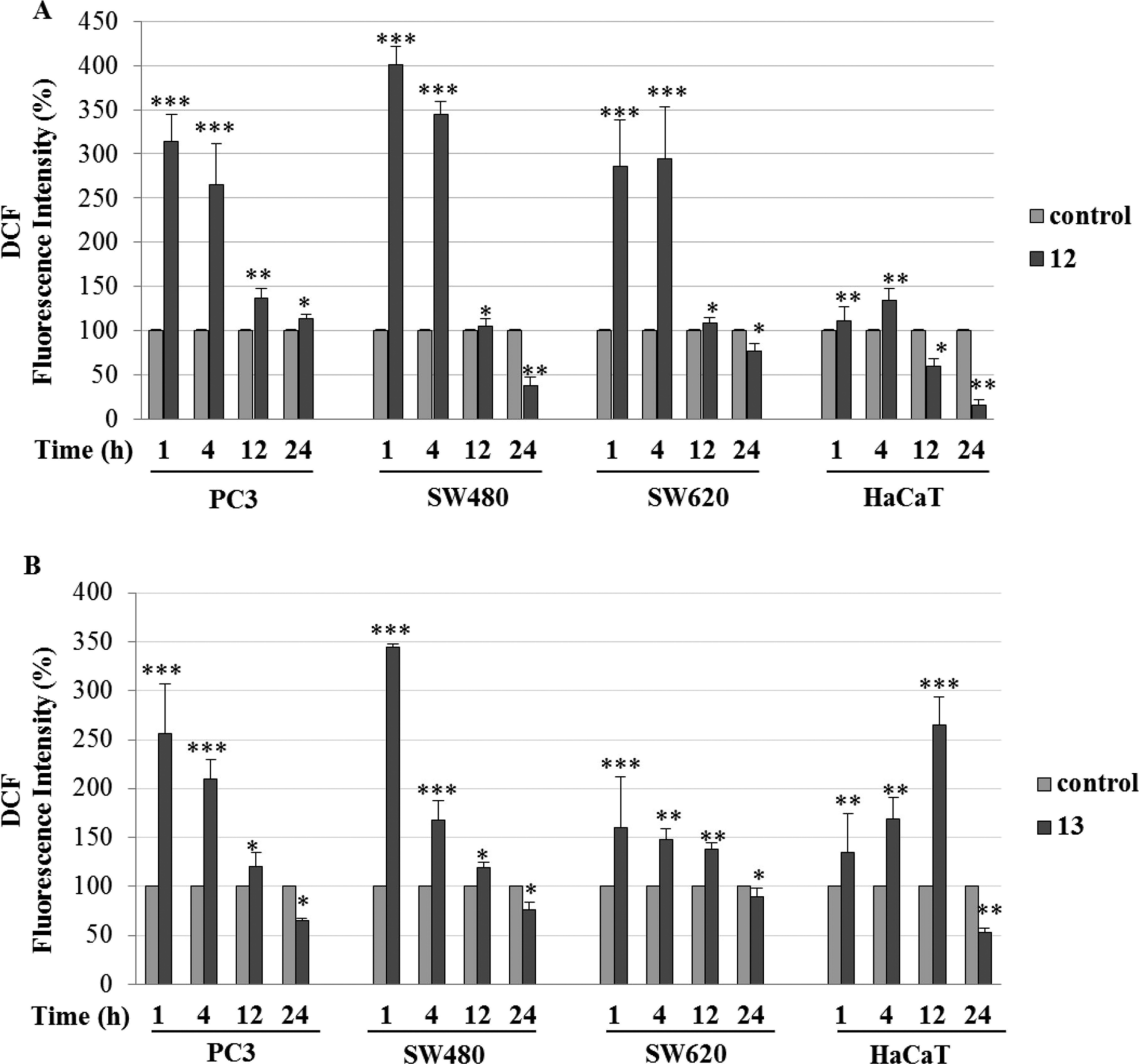
Effects of **LAS**-based bioconjugates **12** and **13** on ROS production. Analysis of ROS generation in PC3, SW480, SW620, and HaCaT cells was performed as described in the Experimental Section. FI of the probe DCF (1 μM) in the presence of compound **12** (A) and compound **13** (B) at their IC_50_ concentrations for 1, 4, 12, or 24 h. The results are expressed as mean ± SD from three experiments, each of them performed in triplicate. ***p ≤ 0.0001, **p ≤ 0.001, *p ≤ 0.01, as compared to the control.

**Figure 6:**
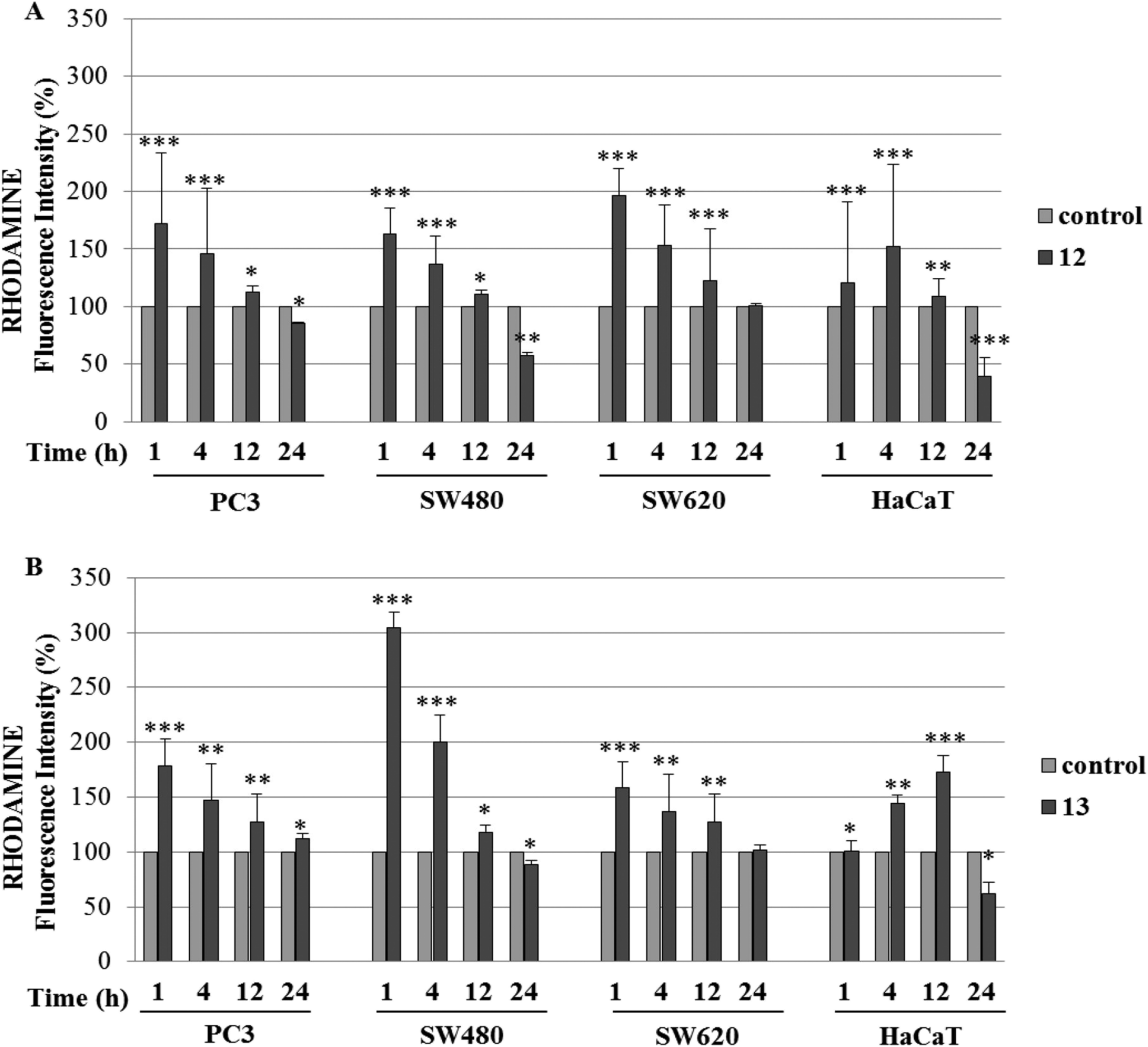
Effects of **LAS**-based bioconjugates **12** and **13** on ROS production. Analysis of ROS generation in PC3, SW480, SW620, and HaCaT cells was performed as described in the Experimental Section. FI of the probe Rhodamine (5 μM) in the presence of compound **12** (A) and compound **13** (B) at their IC_50_ concentrations for 1, 4, 12, or 24 h. The results are expressed as mean ± SD from three experiments, each of them performed in triplicate. ***p ≤ 0.0001, **p ≤ 0.001, *p ≤ 0.01, as compared to the control.

We observed that the dynamics of ROS synthesis following treatment with these compounds differs between cancer and normal cells. In cancer cell lines, the highest level of ROS was found after 1 h, and then it decreased 24 h after treatment (ROS level after 72 h was the same as after 24 h, data not shown) (Figures 5 and 6). In contrast, the amount of ROS in HaCaT cells increased to the highest concentration after 4 and 12 h of treatment for compounds **12** and **13**, respectively, and then decreased (Figures 5 and 6). These differences may result from an altered redox environment in cancer cells compared to normal cells.

It is well known that cancer cells have increased ROS levels to enhance cell signaling essential for cellular transformation and tumorigenesis.^55^ Of note, their antioxidant capacity is also increased to prevent the accumulation of ROS, which may lead to the cell damage. These features make cancer cells more vulnerable to extracellular ROS-generating agents. The elevated ROS levels obtained after treatment with such agents can overcome antioxidant capacity of cancer cells, leading to cell death.^56,57^ In our study, we observed a significantly higher level of ROS in cancer cells compared to that in normal cells after treatment with **LAS** bioconjugate with gemcitabine (compound **12**) (Figures 5A and6A). We can conclude that these elevated amounts of ROS may result in activation of apoptosis/necrosis in cancer cell lines.

Several data indicated that combinations of gemcitabine with other agents can induce cell death through a ROS-mediated mechanism.^58,59^ However, a slightly elevated level of ROS in HaCaT cells treated with compound **12** did not correspond with the high level of late apoptosis observed in these cells. This finding may indicate that compound **12** induces cell death in HaCaT cells by a different mechanism. In contrast, compound **13** indicated a potent nonselective cytotoxic effect on both cancer and normal cells; for **13**, a significantly higher level of ROS was observed in cancer cell lines, especially PC3 and SW480, but also in normal HaCaT cells (Figures 5B and 6B).

## 3 Conclusions

To sum up, nine novel bioconjugates of **LAS** were synthesized. This series included the semisynthetic products obtained exclusively via derivatization of the C1 carboxyl of **LAS**, combining the polyether ionophore biomolecule through covalent bonds with selected oncological drugs (5-fluorouracil, floxuridine, and gemcitabine), betulinic acid, kojic acid, and TPP and ferrocene derivatives. Of note is that our general synthetic pathways are of wide pertinence and might be conveniently applied not only in the preparation of the next generation of bioconjugates of **LAS**, but also other natural polyether ionophores, starting from readily available precursors.

All compounds were evaluated thoroughly for their in vitro antiproliferative activity and selectivity to cancer cells. The most promising semisynthetic products (**LAS** bioconjugate with kojic acid **7**, 5-fluorouracil **8**, gemcitabine **12**, TPP **13**, and ferrocene motif **14**) showed higher anticancer potential against PC3 cancer cells than SW480 and SW620 cancer cells. In general, compounds **7**, **12**, and **13** were identified as the most efficacious anticancer active agents, affecting not only the apoptosis of SW620 cells, but also inducing considerably late apoptosis or necrosis in SW480 and PC3 cells. In addition, compounds **8** and **14** were also strong inducers of late apoptosis and necrosis but only in PC3 cells. Importantly, the most promising bioconjugates of **LAS** significantly reduced the secretion of interleukin 6 (IL-6) in the cancer cells when compared to the control, which may result in the weakening of the IL-6 tumor-promoting effects. Finally, **LAS**-based bioconjugates can induce apoptosis through ROS production, suggesting that ROS, depending on their concentrations, may play a dual role of promoting and inhibiting cancer cell death. Although the obtained products were freely soluble in most organic solvents, they were found to be rather poorly soluble in aqueous buffer of pH 4.0 and pH 7.4. Thus, future work should aim at improvement of the solubility of **LAS**-based bioconjugates in water, which may affect their bioavailability.

## 4 Experimental Section

### 4.1 General Procedures

All commercially available reagents and solvents were purchased from two sources, Merck or Trimen Chemicals S.A. (Poland), and used without further purification. Betulinic acid was purchased from Betulinines (Czech Republic). Detailed descriptions of the general procedures, equipment, measurement parameters, as well as software can be found in the Supporting Information.

### 4.2 Isolation of LAS and Synthesis of Key Precursors **1–5**

**LAS** in acid form was prepared conveniently by isolation of its sodium salt from commercially available veterinary premix AVATEC, followed by acidic extraction with H_2_SO_4_ (pH = 1.0), according to a previously reported protocol.^22^ All three precursors of 5-fluorouracil (compounds **1–3**) were obtained on the basis of the one-pot base silylation/nucleoside coupling procedure published by Liu and co-workers,^60^ using BSA as the silylating agent and I_2_ as the Lewis acid. On the other hand, compound **4** was formed in the reaction of kojic acid with thionyl chloride, as recently reported by Agyemang and Murelli,^32^ while 6-bromohexyl ester at the C1 position of **LAS** (compound **5**) was resynthesized on the basis of the procedure published by us previously.^33^ The NMR data concerning literature-known products (compounds **1**, **4,** and **5**) were in good agreement with those found in the reference literature.^32−34^^1^H NMR, ^13^C NMR, and ^19^F NMR spectra of newly synthesized precursors **2** and **3** can be found in the Supporting Information (Figures S1–S6).

#### 4.2.1 1-(6-Bromohexyl)-5-fluorouracil **2**

Yield: 4.65 g, 52%. Isolated as a cream amorphous solid, >95% pure by NMR and a single spot by TLC; *R*_*f*_: 0.57 in 60% EtOAc/*n*-hexane. UV active; ^1^H NMR (403 MHz, CDCl_3_): δ 9.87 (s, 1H), 7.26 (d, *J* = 5.5 Hz, 1H), 3.82–3.63 (m, 2H), 3.41 (t, *J* = 6.7 Hz, 2H), 1.94–1.81 (m, 2H), 1.79–1.66 (m, 2H), 1.58–1.44 (m, 2H), 1.44–1.31 (m, 2H) ppm; ^13^C NMR (101 MHz, CDCl_3_): δ 157.4, 157.1, 149.6, 141.6, 139.3, 128.6, 128.3, 48.9, 33.5, 32.3, 28.6, 27.5, 25.4 ppm; ^19^F NMR (282 MHz, CDCl_3_): δ −166.16, −166.17, −166.18 ppm; ESI MS (*m*/*z*): [M + H]^+^ calcd for C_10_H_15_BrFN_2_O_2_^+^, 293.0; found, 293; HRMS (ESI^+^) *m*/*z*: [M + H]^+^ calcd for C_10_H_15_BrFN_2_O_2_^+^, 293.0301; found, 293.0297.

#### 4.2.2 1-(2-(2-Bromoethoxy)ethyl)-5-fluorouracil **3**

Yield: 1.38 g, 27%. Isolated as a cream amorphous solid, >95% pure by NMR and a single spot by TLC; *R*_*f*_: 0.80 in 8% MeOH/CHCl_3_. UV active; ^1^H NMR (403 MHz, CDCl_3_): δ 9.62 (s, 1H), 7.49 (d, *J* = 5.7 Hz, 1H), 3.95 (dd, *J* = 5.2, 4.2 Hz, 2H), 3.84–3.77 (m, 2H), 3.75 (dd, *J* = 5.2, 4.2 Hz, 2H), 3.51–3.42 (m, 2H) ppm; ^13^C NMR (101 MHz, CDCl_3_): δ 157.4, 157.1, 149.6, 141.2, 138.8, 130.4, 130.1, 70.9, 68.7, 48.5, 30.3 ppm; ^19^F NMR (282 MHz, CDCl_3_): δ −167.43, −167.44, −167.46 ppm; ESI MS (*m*/*z*): [M + H]^+^ calcd for C_8_H_11_BrFN_2_O_3_^+^, 281.0; found, 281; HRMS (ESI^+^) *m*/*z*: [M + H]^+^ calcd for C_8_H_11_BrFN_2_O_3_^+^, 280.9937; found, 280.9934.

### 4.3 General Procedure for the Preparation of **LAS** Conjugates **6–10** and **13–14**

A mixture of **LAS** or betulinic acid (1.0 equiv), DBU (1.2 equiv), and the corresponding bromide/chloride (2.0 equiv) in the respective anhydrous aprotic solvent was heated for a few hours; please see Scheme 2 for more details. After that, the reaction mixture was concentrated under reduced pressure. Purification on silica gel using the CombiFlash system gave the pure products of the reaction **6–10** and **13–14** (12–36% yield) as oils. The oils were then diluted in *n*-pentane and evaporated to dryness three times to form amorphous solids in most cases. The NMR spectra of compounds **6–10** and **13–14** are included in the Supporting Information (Figures S7–S19 and S26–S30).

#### 4.3.1 **LAS**–Betulinic Acid Conjugate **6**

Yield: 34 mg, 20%. Isolated as a white amorphous solid, >95% pure by NMR and a single spot by TLC; UV active; ^1^H NMR (403 MHz, CDCl_3_): δ 11.45 (s, 1H), 7.15 (dd, *J* = 7.6, 2.0 Hz, 1H), 6.67 (dd, *J* = 7.6, 3.4 Hz, 1H), 4.72 (d, *J* = 2.1 Hz, 1H), 4.59 (dd, *J* = 2.0, 1.4 Hz, 1H), 4.39 (t, *J* = 6.9 Hz, 2H), 4.07 (ddt, *J* = 24.0, 10.9, 6.6 Hz, 2H), 3.99–3.85 (m, 1H), 3.82 (dd, *J* = 10.0, 3.9 Hz, 1H), 3.79–3.72 (m, 1H), 3.45 (dd, *J* = 11.4, 1.9 Hz, 1H), 3.30 (s, 1H), 3.17 (dd, *J* = 11.0, 4.7 Hz, 1H), 3.00 (td, *J* = 10.8, 4.5 Hz, 1H), 2.92 (ddt, *J* = 17.1, 8.5, 4.5 Hz, 2H), 2.84–2.75 (m, 1H), 2.66–2.52 (m, 1H), 2.29–2.16 (m, 4H), 2.14–0.50 (m, 88H) ppm; ^13^C NMR (101 MHz, CDCl_3_): δ 215.1, 176.1, 171.9, 160.7, 150.6, 143.3, 134.9, 124.1, 121.6, 111.3, 109.5, 85.8, 85.0, 78.9, 77.0, 73.9, 71.5, 70.4, 65.5, 63.7, 56.5, 55.3, 54.8, 50.5, 49.35, 49.32, 47.0, 42.3, 40.6, 38.8, 38.7, 38.2, 37.1, 37.0, 36.3, 35.1, 34.3, 34.2, 34.0, 32.1, 30.60, 30.59, 30.0, 29.7, 29.6, 29.4, 28.6, 28.5, 27.9, 27.4, 25.8, 25.7, 25.5, 21.0, 20.9, 19.3, 18.3, 18.2, 16.1, 16.01, 15.96, 15.9, 15.3, 14.6, 14.1, 13.5, 12.7, 12.3, 8.5, 6.4 ppm; FT-IR (KBr tablet): 3449 (m, br), 3070 (m), 2958 (s), 2928 (s), 2871 (s), 1719 (s), 1654 (s), 1615 (m), 1582 (w), 1458 (s), 1412 (s), 1389 (s), 1378 (s) cm^–1^; ESI MS (*m*/*z*): [M + Na]^+^ calcd for C_70_H_112_NaO_11_^+^, 1151.8; found, 1151.8; HRMS (ESI^+^) *m*/*z*: [M + Na]^+^ calcd for C_70_H_112_NaO_11_^+^, 1151.8102; found, 1151.8077.

#### 4.3.2 **LAS**–Kojic Acid Conjugate **7**

Yield: 32 mg, 12%. Isolated as a white amorphous solid, >95% pure by NMR and a single spot by TLC; *R*_*f*_: 0.37 in 50% EtOAc/*n*-hexane. UV active; ^1^H NMR (401 MHz, CD_2_Cl_2_): δ 11.07 (s, 1H), 7.96 (s, 1H), 7.21 (dd, *J* = 7.6, 0.6 Hz, 1H), 6.71 (d, *J* = 7.6 Hz, 1H), 6.58 (s, 1H), 5.35–5.30 (m, 1H), 5.29–5.23 (m, 1H), 3.99 (dd, *J* = 9.5, 1.5 Hz, 1H), 3.89 (dd, *J* = 10.2, 4.0 Hz, 1H), 3.73 (q, *J* = 6.9 Hz, 1H), 3.41 (dd, *J* = 11.7, 2.0 Hz, 1H), 3.04–2.80 (m, 3H), 2.70 (dt, *J* = 10.4, 3.5 Hz, 1H), 2.26–2.13 (m, 4H), 1.90–0.70 (m, 39H) ppm; ^13^C NMR (101 MHz, CD_2_Cl_2_): δ 214.1, 174.3, 171.7, 162.8, 161.6, 146.5, 144.4, 138.9, 136.3, 124.8, 122.6, 112.7, 111.0, 86.9, 85.4, 78.0, 74.0, 71.8, 70.7, 63.0, 55.0, 49.1, 39.3, 37.2, 35.3, 35.2, 34.7, 31.4, 31.2, 30.2, 21.3, 17.6, 16.1, 15.9, 14.6, 14.0, 13.1, 12.6, 9.1, 6.8 ppm; FT-IR (KBr tablet): 3440 (s, br), 3098 (m, br), 2963 (s), 2935 (s), 2877 (s), 1739 (m), 1714 (s), 1652 (s), 1631 (s), 1615 (s), 1595 (m), 1582 (m), 1493 (w), 1458 (s), 1410 (s), 1381 (s) cm^–1^; ESI MS (*m*/*z*): [M + Na]^+^ calcd for C_40_H_58_NaO_11_^+^, 737.4; found, 737; HRMS (ESI^+^) *m*/*z*: [M + Na]^+^ calcd for C_40_H_58_NaO_11_^+^, 737.3877; found, 737.3865.

#### 4.3.3 **LAS**–5-Fluorouracil Conjugate **8**

Yield: 230 mg, 22%. Isolated as a white amorphous solid, >95% pure by NMR and a single spot by TLC; *R*_*f*_: 0.50 in 60% EtOAc/*n*-hexane. UV active; ^1^H NMR (403 MHz, CDCl_3_): δ 11.44 (s, 1H), 9.79 (d, *J* = 3.8 Hz, 1H), 7.60 (d, *J* = 5.5 Hz, 1H), 7.16 (d, *J* = 7.6 Hz, 1H), 6.65 (d, *J* = 7.6 Hz, 1H), 4.46 (tdd, *J* = 16.5, 11.1, 5.7 Hz, 2H), 4.02 (dd, *J* = 9.4, 0.8 Hz, 1H), 3.93–3.71 (m, 3H), 3.42 (dd, *J* = 11.8, 1.4 Hz, 1H), 3.35 (s, 1H), 3.17 (ddd, *J* = 12.6, 10.7, 5.5 Hz, 1H), 2.92 (ddd, *J* = 14.2, 9.1, 7.0 Hz, 1H), 2.80–2.59 (m, 2H), 2.21 (s, 3H), 2.10–0.60 (m, 43H) ppm; ^13^C NMR (101 MHz, CDCl_3_): δ 214.4, 171.9, 160.8, 157.4, 157.2, 149.8, 143.1, 141.7, 139.4, 135.0, 129.1, 128.8, 124.1, 121.5, 111.2, 86.5, 84.5, 77.4, 72.5, 71.1, 70.3, 64.7, 54.6, 48.9, 48.6, 38.5, 36.1, 34.8, 34.2, 33.8, 30.7, 30.4, 29.6, 29.5, 25.7, 25.4, 20.4, 16.9, 15.8, 15.6, 14.2, 13.5, 12.7, 8.8, 6.4 ppm; ^19^F NMR (282 MHz, CDCl_3_): δ −166.54, −166.56, −166.57 ppm; FT-IR (KBr tablet): 3464 (s, br), 3189 (m, br), 3064 (s), 2964 (s), 2936 (s), 2878 (s), 1704 (s), 1660 (s), 1616 (m), 1583 (w), 1459 (s), 1412 (s), 1379 (s) cm^–1^; ESI MS (*m*/*z*): [M + Na]^+^ calcd for C_42_H_63_FN_2_NaO_10_^+^, 797.4; found, 797.4; HRMS (ESI^+^) *m*/*z*: [M + Na]^+^ calcd for C_42_H_63_FN_2_NaO_10_^+^, 797.4364; found, 797.4351.

#### 4.3.4 **LAS**–5-Fluorouracil Conjugate **9**

Yield: 400 mg, 36%. Isolated as a white amorphous solid, >95% pure by NMR and a single spot by TLC; *R*_*f*_: 0.57 in 66% EtOAc/*n*-hexane. UV active; ^1^H NMR (403 MHz, CDCl_3_): δ 11.49 (s, 1H), 9.82 (d, *J* = 3.6 Hz, 1H), 7.39 (d, *J* = 5.5 Hz, 1H), 7.15 (d, *J* = 7.6 Hz, 1H), 6.65 (d, *J* = 7.6 Hz, 1H), 4.41 (pd, *J* = 10.9, 6.3 Hz, 2H), 3.90 (dd, *J* = 9.4, 0.7 Hz, 1H), 3.84 (dd, *J* = 10.1, 3.1 Hz, 1H), 3.80–3.61 (m, 3H), 3.44 (dd, *J* = 11.5, 1.3 Hz, 1H), 3.23 (s, 3H), 3.03–2.82 (m, 3H), 2.75–2.64 (m, 1H), 2.21 (s, 3H), 2.00–0.60 (m, 44H) ppm; ^13^C NMR (101 MHz, CDCl_3_): δ 214.8, 172.0, 160.8, 157.4, 157.1, 149.8, 143.1, 141.7, 139.3, 134.9, 128.6, 128.3, 124.1, 121.7, 111.3, 86.1, 84.6, 77.4, 73.8, 71.3, 70.3, 65.4, 54.8, 49.2, 48.8, 38.8, 36.3, 35.1, 34.1, 33.6, 30.7, 30.2, 29.6, 29.4, 28.7, 28.4, 26.0, 25.8, 20.6, 17.4, 15.8, 14.2, 13.4, 12.7, 11.9, 8.7, 6.4 ppm; ^19^F NMR (282 MHz, CDCl_3_): δ −166.60, −166.61, −166.63 ppm; FT-IR (KBr tablet): 3466 (m, br), 3188 (m, br), 3063 (m), 2961 (s), 2932 (s), 2876 (s), 1710 (s), 1660 (s), 1616 (m), 1583 (w), 1460 (s), 1412 (s), 1381 (s) cm^–1^; ESI MS (*m*/*z*): [M + Na]^+^ calcd for C_44_H_67_FN_2_NaO_10_^+^, 825.5; found, 825.5; HRMS (ESI^+^) *m*/*z*: [M + Na]^+^ calcd for C_44_H_67_FN_2_NaO_10_^+^, 825.4677; found, 825.4658.

#### 4.3.5 **LAS**–5-Fluorouracil Conjugate **10**

Yield: 330 mg, 25%. Isolated as a white amorphous solid, >95% pure by NMR and a single spot by TLC; *R*_*f*_: 0.60 in 60% EtOAc/*n*-hexane. UV active; ^1^H NMR (403 MHz, CDCl_3_): δ 11.19 (s, 1H), 9.44 (s, 1H), 7.49 (d, *J* = 5.7 Hz, 1H), 7.17 (d, *J* = 7.6 Hz, 1H), 6.67 (d, *J* = 7.6 Hz, 1H), 4.64–4.48 (m, 2H), 4.04–3.92 (m, 2H), 3.91–3.71 (m, 6H), 3.42 (dd, *J* = 11.6, 1.5 Hz, 1H), 3.06 (ddd, *J* = 13.2, 10.1, 5.8 Hz, 1H), 2.90 (dq, *J* = 9.7, 7.0 Hz, 1H), 2.76 (ddd, *J* = 12.9, 10.3, 4.7 Hz, 1H), 2.66 (dt, *J* = 10.3, 3.0 Hz, 1H), 2.21 (s, 3H), 2.10–0.60 (m, 40H) ppm; ^13^C NMR (101 MHz, CDCl_3_): δ 214.5, 171.5, 160.5, 157.2, 156.9, 149.7, 143.2, 141.3, 139.0, 135.2, 130.1, 129.7, 124.2, 121.7, 111.2, 86.2, 84.7, 77.2, 73.2, 71.4, 70.4, 69.0, 68.5, 63.9, 54.8, 48.9, 48.6, 39.0, 36.1, 35.1, 34.1, 33.9, 30.7, 30.2, 29.5, 20.6, 17.3, 15.8, 14.1, 13.6, 12.7, 12.3, 8.6, 6.4 ppm, one signal overlapped; ^19^F NMR (282 MHz, CDCl_3_): δ −167.35, −167.36 ppm; FT-IR (KBr tablet): 3462 (m, br), 3190 (m, br), 3067 (m), 2965 (s), 2938 (s), 2879 (s), 1712 (s), 1664 (s), 1615 (m), 1582 (w), 1459 (s), 1444 (s), 1412 (s), 1380 (s) cm^–1^; ESI MS (*m*/*z*): [M + Na]^+^ calcd for C_42_H_63_FN_2_NaO_11_^+^, 813.4; found, 813; HRMS (ESI^+^) *m*/*z*: [M + Na]^+^ calcd for C_42_H_63_FN_2_NaO_11_^+^, 813.4314; found, 813.4297.

#### 4.3.6 **LAS**–TPP Conjugate **13**

Yield: 341 mg, 23%. Isolated as a cream amorphous solid, >95% pure by NMR and a single spot by TLC; *R*_*f*_: 0.38 in 33% acetone/CH_2_Cl_2_. UV active; ^1^H NMR (400 MHz, CD_3_CN): δ 10.82 (s, 1H), 7.93–7.74 (m, 9H), 7.74–7.60 (m, 6H), 7.21 (dd, *J* = 7.6, 0.6 Hz, 1H), 6.72 (d, *J* = 7.6 Hz, 1H), 4.66–4.58 (m, 1H), 4.53–4.45 (m, 1H), 4.12 (s, 1H), 3.88 (dd, *J* = 10.1, 3.5 Hz, 2H), 3.64 (dd, *J* = 13.6, 6.7 Hz, 1H), 3.61–3.36 (m, 5H), 3.17–3.09 (m, 1H), 2.98–2.90 (m, 1H), 2.77–2.71 (m, 1H), 2.71–2.62 (m, 1H), 2.26–2.01 (m, 7H); 2.00–0.60 (m, 34H) ppm; ^13^C NMR (101 MHz, CD_3_CN): δ 214.8, 172.3, 160.3, 144.2, 136.05, 136.02, 135.98, 135.7, 134.69, 134.67, 134.59, 134.57, 131.2, 131.1, 124.7, 122.7, 119.38, 119.35, 118.51, 118.49, 113.4, 87.2, 85.0, 77.9, 73.6, 71.8, 70.8, 65.6, 65.4, 54.9, 48.7, 39.2, 37.4, 35.6, 34.9, 34.7, 31.7, 31.1, 30.3, 22.50, 22.47, 21.1, 20.2, 19.7, 17.4, 16.0, 15.5, 14.6, 13.9, 13.1, 12.6, 9.1, 6.7 ppm, one signal overlapped; ^31^P NMR (162 MHz, CD_3_CN): δ 25.39 ppm; FT-IR (KBr tablet): 3401 (s, br), 3056 (m), 2963 (s), 2933 (s), 2876 (s), 1726 (s), 1712 (s), 1654 (s), 1615 (m), 1587 (m), 1580 (m), 1486 (m), 1459 (s), 1439 (s), 1411 (s), 1379 (s) cm^–1^; ESI MS (*m*/*z*): [M–Br]^+^ calcd for C_55_H_74_O_8_P^+^, 893.5; found, 894; HRMS (ESI^+^): *m*/*z*: [M–Br]^+^ calcd for C_55_H_74_O_8_P^+^, 893.5116; found, 893.5126.

#### 4.3.7 **LAS**–Ferrocene Conjugate **14**

Yield: 36 mg, 21%. Isolated as an orange oil, >95% pure by NMR and a single spot by TLC; *R*_*f*_: 0.64 in 33% EtOAc/*n*-hexane. UV active; ^1^H NMR (401 MHz, CDCl_3_): δ 11.46 (s, 1H), 7.15 (d, *J* = 7.6 Hz, 1H), 6.66 (dd, *J* = 7.6, 2.6 Hz, 1H), 4.81–4.75 (m, 2H), 4.51–4.46 (m, 2H), 4.42 (td, *J* = 6.7, 1.3 Hz, 2H), 4.18 (s, 5H), 3.97–3.87 (m, 1H), 3.77 (ddt, *J* = 13.5, 7.3, 4.3 Hz, 2H), 3.43 (td, *J* = 11.5, 2.0 Hz, 1H), 3.02–2.83 (m, 3H), 2.78 (dt, *J* = 10.4, 3.6 Hz, 1H), 2.73 (t, *J* = 7.4 Hz, 2H), 2.34–2.10 (m, 4H), 2.10–0.60 (m, 44H) ppm; ^13^C NMR (101 MHz, CDCl_3_): δ 215.2, 204.1, 171.9, 160.7, 143.3, 134.9, 124.1, 121.6, 111.4, 85.7, 85.0, 77.1, 76.5, 74.1, 72.1, 71.7, 70.4, 69.7, 69.3, 65.5, 55.0, 49.3, 39.4, 36.4, 35.3, 34.2, 34.1, 30.6, 30.0, 29.3, 28.6, 26.0, 24.1, 21.1, 18.3, 16.1, 15.9, 14.1, 13.5, 12.7, 12.3, 12.2, 8.4, 6.4 ppm; FT-IR (KBr tablet): 3442 (s, br), 3096 (m), 2961 (s), 2935 (s), 2877 (s), 1712 (s), 1666 (s), 1655 (s), 1615 (s), 1582 (m), 1457 (s), 1412 (s), 1396 (s), 1379 (s) cm^–1^; ESI MS (*m*/*z*): [M + Na]^+^ calcd for C_50_H_72_FeNaO_9_^+^, 895.4; found, 896; HRMS (ESI^+^) *m*/*z*: [M + Na]^+^ calcd for C_50_H_72_FeNaO_9_^+^, 895.4423; found, 895.4431.

### 4.4 General Procedure for the Preparation of **LAS** Conjugates **11–12**

To a mixture of **LAS** (1.0 equiv) in anhydrous DMF at 0 °C, the following compounds were added: DCC (1.5 equiv), PPy (0.5 equiv), respective alcohol (8.0 equiv), and catalytic pTSA. The mixture was allowed to warm to room temperature and stirred for further 3 days. After that, the reaction mixture was concentrated under reduced pressure. Purification on silica gel using the CombiFlash system gave the pure products of the reaction **11–12** (11–24% yield) as clear oils. The oils were then diluted in *n*-pentane and evaporated to dryness three times to form white amorphous solids. The NMR spectra of compounds **11–12** are included in the Supporting Information (Figures S20–S25).

#### 4.4.1 **LAS**–Floxuridine Conjugate **11**

Yield: 32 mg, 11%. Isolated as a white amorphous solid, >95% pure by NMR and a single spot by TLC; *R*_*f*_: 0.69 in 100% EtOAc. UV active; ^1^H NMR (400 MHz, CDCl_3_): δ 10.96 (s, 1H), 8.91 (s, 1H), 7.47 (d, *J* = 6.0 Hz, 1H), 7.17 (d, *J* = 7.7 Hz, 1H), 6.65 (d, *J* = 7.6 Hz, 1H), 6.25 (td, *J* = 6.5, 1.4 Hz, 1H), 4.77 (dd, *J* = 11.9, 5.2 Hz, 1H), 4.62–4.54 (m, 2H), 4.27 (dd, *J* = 8.9, 4.4 Hz, 1H), 4.05 (dd, *J* = 9.6, 1.3 Hz, 1H), 3.85 (dd, *J* = 10.2, 3.4 Hz, 1H), 3.77 (dd, *J* = 13.3, 6.5 Hz, 1H), 3.70 (s, 1H), 3.42 (dd, *J* = 11.6, 1.9 Hz, 1H), 3.15 (ddd, *J* = 12.8, 10.5, 5.5 Hz, 1H), 2.92 (ddd, *J* = 14.1, 9.6, 7.0 Hz, 1H), 2.76 (ddd, *J* = 12.8, 10.4, 5.6 Hz, 1H), 2.67 (dt, *J* = 10.6, 3.2 Hz, 1H), 2.49 (ddd, *J* = 13.8, 6.3, 4.0 Hz, 1H), 2.30–2.08 (m, 6H), 2.05–0.60 (m, 37H) ppm; ^13^C NMR (101 MHz, CDCl_3_): δ 214.6, 171.5, 160.4, 156.7, 156.5, 148.5, 142.7, 141.7, 139.3, 135.3, 124.4, 124.0, 123.7, 121.8, 111.1, 86.8, 85.7, 84.7, 83.9, 77.2, 72.6, 70.9, 70.7, 70.6, 63.9, 54.5, 48.8, 39.9, 38.5, 36.3, 34.5, 34.1, 33.7, 30.7, 30.1, 29.7, 20.2, 16.7, 15.9, 15.5, 14.1, 13.5, 12.7, 12.6, 8.9, 6.4 ppm; ^19^F NMR (283 MHz, CDCl_3_): δ −164.65, −164.68 ppm; FT-IR (KBr tablet): 3435 (s, br), 3206 (m, br), 3085 (m, br), 3028 (m), 2964 (s), 2937 (s), 2879 (s), 1709 (s), 1663 (s), 1615 (m), 1582 (w), 1459 (s), 1410 (s), 1381 (s) cm^–1^; ESI MS (*m*/*z*): [M + Na]^+^ calcd for C_43_H_63_FN_2_NaO_12_^+^, 841.4; found, 841; HRMS (ESI^+^) *m*/*z*: [M + Na]^+^ calcd for C_43_H_63_FN_2_NaO_12_^+^, 841.4263; found, 841.4253.

#### 4.4.2 **LAS**–Gemcitabine Conjugate **12**

Yield: 145 mg, 24%. Isolated as a white amorphous solid, >95% pure by NMR and a single spot by TLC; *R*_*f*_: 0.65 in 50% EtOAc/acetone. UV active; ^1^H NMR (400 MHz, CDCl_3_): δ 10.97 (s, 1H), 7.23 (d, *J* = 7.5 Hz, 1H), 7.17 (dd, *J* = 7.6, 0.5 Hz, 1H), 6.64 (d, *J* = 7.6 Hz, 1H), 6.34 (t, *J* = 8.0 Hz, 1H), 6.05 (s, 2H), 5.62 (d, *J* = 7.5 Hz, 1H), 4.94 (dd, *J* = 11.9, 3.4 Hz, 1H), 4.53 (dd, *J* = 12.0, 3.7 Hz, 1H), 4.35–4.22 (m, 2H), 3.97 (d, *J* = 9.4 Hz, 1H), 3.87 (dd, *J* = 13.6, 6.7 Hz, 1H), 3.81 (dd, *J* = 10.2, 3.0 Hz, 1H), 3.67 (s, 1H), 3.42 (dd, *J* = 11.6, 1.0 Hz, 1H), 3.02 (ddd, *J* = 12.4, 8.4, 6.4 Hz, 1H), 2.94–2.83 (m, 1H), 2.82–2.73 (m, 1H), 2.70–2.63 (m, 1H), 2.24–2.14 (m, 4H), 2.05–0.60 (m, 38H) ppm; ^13^C NMR (101 MHz, CDCl_3_): δ 215.0, 171.2, 165.6, 160.5, 155.5, 142.9, 140.8, 135.2, 124.4, 121.9, 121.7, 111.1, 95.3, 86.5, 84.3, 78.6, 78.5, 77.2, 77.0, 72.9, 71.1, 70.8, 62.8, 54.7, 49.0, 38.5, 36.0, 34.7, 34.1, 33.3, 30.7, 29.63, 29.57, 20.1, 16.9, 15.9, 15.6, 14.2, 13.3, 12.7, 12.3, 8.8, 6.4 ppm; ^19^F NMR (283 MHz, CDCl_3_): δ −117.68, −118.55, −120.96, −121.90 ppm; FT-IR (KBr tablet): 3419 (s, br), 3352 (s, br), 3213 (s, br), 3108 (m), 3101 (m), 2965 (s), 2937 (s), 2879 (s), 1732 (m), 1707 (s), 1654 (s), 1616 (s), 1584 (m), 1523 (s), 1494 (s), 1459 (s), 1409 (s), 1380 (s) cm^–1^; ESI MS (*m*/*z*): [M + Na]^+^ calcd for C_43_H_63_F_2_N_3_NaO_11_^+^, 858.4; found, 858; HRMS (ESI^+^) *m*/*z*: [M + Na]^+^ calcd for C_43_H_63_F_2_N_3_NaO_11_^+^, 858.4328; found, 858.4320.

### 4.5 Cell Line and Culture

The human cell lines SW480 (primary colon cancer), SW620 (lymph node metastatic colon cancer from the same patient as primary cancer cells), PC3 (metastatic prostate cancer), and HaCaT (immortalized keratinocytes) were obtained from the American Type Culture Collection (ATCC, Rockville, USA). The SW480 and SW620 cells were grown in MEM (ThermoSci, USA), PC3 in RPMI 1640, and HaCaT in DMEM High Glucose (Biowest SAS, France) supplemented with 10% foetal bovine serum (FBS), HEPES (20 mM), and antibiotics (100 U mL^–1^ of penicillin and 100 μg mL^–1^ of streptomycin). The cells were incubated in a humidified incubator at 37 °C/5% CO_2_, until 80–90% confluence was reached.

### 4.6 MTT Assay

The cell viability was assessed by using of MTT salt [3-(4,5-dimethylthiazol-2-yl)-2,5-diphenyltetrazolium bromide] converted by mitochondrial dehydrogenase, occurring in living cells. The cells were seeded in 96-well plates at a density of 1 × 10^4^ cells per well and allowed to adhere for 24 h at 37 °C in a CO_2_ humidified incubator. Then, the medium was removed and a fresh medium with various concentrations of tested compounds (from 10 to 120 μM) was added. The untreated cells were used as the control.

After 72 h incubation, the medium was replaced with 200 μL per well of free serum medium containing 0.5 mg mL^–1^ MTT and incubated for 4 h at 37 °C in a CO_2_ humidified incubator. Subsequently, the medium was removed and dimethyl sulfoxide with isopropanol (1:1) was added to dissolve the formazan crystals. The optical density was measured using a UVM 340 reader (ASYS Hitech GmbH, Austria) at a wavelength of 570 nm. The experiments were repeated three times. The cell viability was calculated as the percent of MTT reduced in treated cells versus control cells (untreated cells). The number of viable cells cultured without tested compounds was assumed as 100%. The decreased relative MTT level indicates decreased cell viability. The IC_50_ values were estimated using CompuSyn version 1.0.

### 4.7 Annexin V-FITC/PI Binding Assay

The SW480, SW620, PC3, and HaCaT cells were cultured and harvested under the conditions mentioned in the Section 4.5 and seeded in 12-well plates (1 × 10^5^ cells per well). After 24 h pre-incubation, the cells were treated with the tested compounds at IC_50_ concentrations and incubated for 72 h. The apoptotic effect was performed using the Annexin V-FITC/propidium iodide (PI) apoptosis assay kit (Becton Dickinson, Pharmingen), according to the manufacturer’s instructions, and analyzed by flow cytometry (Becton Dickinson). The cells which were Annexin V-FITC-positive and PI-negative were identified as early apoptotic and both Annexin V-FITC- and PI-positive as late apoptotic or necrotic. The experiment was repeated three times.

### 4.8 Interleukin-6 Assay

The level of interleukin-6 (IL-6) in SW480, SW620, and PC3 cell lines was measured by commercial human IL-6 ELISA kits Diaclon SAS (Besancon Cedex, France). The cells were treated with IC_50_ concentrations of the tested compounds for 72 h. The untreated cells were used as the control. The IL-6 level in a cell culture supernatant was measured using an enzyme-linked immunosorbent assay, in accordance with the manufacturer’s instruction. The experiment was repeated three times.

### 4.9 ROS Detection—DCFH-DA and DHR-123 Assay

ROS generation was assessed by the spectrofluorometric method using the 2′,7′-dichlorodihydrofluorescein diacetate (DCFH-DA) or dihydrorhodamine 123 (DHR-123). The method is based on the ROS-dependent oxidation of the compounds to fluorescent dichlorofluorescein (DCF) or rhodamine-123, respectively.^61^ PC3, SW480, SW620, and HaCaT were seeded on to 96-well plates (5 × 10^4^ cells per well) and allowed to adhere for 24 h. Then, cells were rinsed with PBS and incubated with DCFH-DA (5 μM) or DHR-123 (1 μM) for 30 min at 37 °C in the dark. Thereafter, cells were rinsed with PBS and treated for 1, 4, 12, 24, and 72 h at 37 °C with red phenol free culture medium containing compound **12** or **13** at their IC_50_ concentrations to observe the level of ROS. A sample with H_2_O_2_ (1.5 mM) was a positive control, and a sample without any reagent was a negative control. Maximum excitation and emission spectra for DCF were 492 and 527 nm, and those for rhodamine-123 were 500 and 536 nm, respectively. The generation of H_2_O_2_ was measured by Microplate Spectrofluorometer BioTek Synergy (BioTek Instruments, USA) and expressed as fluorescence intensity (FI). Values from three experiments performed in triplicate were analyzed.

### 4.10 Statistical Analysis

The statistical calculation was performed using Statistica 13.1 (StatSoft, Inc, USA) program. The quantitative comparisons were made using Student’s *t*-test. The IC_50_ values were estimated by CompuSyn version 1.0. Results of all presented experiments were expressed as the mean ± SD and considered statistically significant at *p* < 0.05.

## Supporting Information Available

Detailed descriptions of the general procedures, equipment, measurement parameters, as well as software; ^1^H NMR, ^13^C NMR, ^19^F NMR, or ^31^P NMR spectra of newly synthesized precursors **2** and **3**; and all **LAS**-based bioconjugates **6–14** (PDF)
