# Supplementary material for: Synthesis of Lasalocid-Based Bioconjugates and Evaluation of Their Anticancer Activity
Source: ACS Omega. 2022 Jan 7;7(2):1943–55. doi: 10.1021/acsomega.1c05434 (PMC8771711; doi:10.1021/acsomega.1c05434)
Supplement: Supplementary file 1 — ao1c05434_si_001.pdf [file ao1c05434_si_001.pdf]

Supplementary Material

Synthesis of lasalocid-based bioconjugates and evaluation of their anticancer activity

Michał Antoszczak [a,*], *Dagmara Otto-Ślusarczyk* [b], *Marta Kordylas* [a],

Marta Struga [b] *and Adam Huczyński* [a]

[a] Department of Medical Chemistry, Faculty of Chemistry, Adam Mickiewicz University, Uniwersytetu Poznańskiego 8, 61–614 Poznań, Poland

[b] Chair and Department of Biochemistry, Faculty of Medicine, Medical University of Warsaw, Banacha 1, 02–097 Warsaw, Poland

Index

General procedures ....– S2 –

Spectroscopic analysis of newly synthesized compounds ....– S3 –

General procedures

All reagents and solvents were obtained from Merck or Trimen Chemicals S.A. (Poland), and were used as received without further purification. $CDCl_3$, CD_2Cl_2 and CD_3CN spectral grade solvent was stored over 3Å molecular sieves for several days. All manipulations were carried out under nitrogen atmosphere in oven-dried glassware. Reaction mixtures were stirred using teflon-coated magnetic stir bars. Reaction mixtures were monitored by thin layer chromatography (TLC) using aluminium-backed plates (Merck $60F_{254}$). TLC plates were visualized by UV-light (254 nm), after treated with phosphomolybdic acid (PMA, 5% in absolute EtOH) and gentle heating. Products of the reactions were purified using CombiFlash Rf^+ Lumen Flash Chromatography System (Teledyne Isco) with integrated ELS and UV detectors. All solvents used in flash chromatography were of HPLC grade (Merck), and were used as received. Solvents were removed using a rotary evaporator.

NMR spectra were recorded on a Varian 400 (^{1}H NMR at 400 MHz, ^{13}C NMR at 101 MHz, ^{19}F NMR at 282 MHz, and ^{31}P NMR at 162 MHz) magnetic resonance spectrometer. ^{1}H NMR spectra are reported in chemical shifts downfield from TMS using the respective residual solvent peak as internal standard ($CDCl_3$ δ 7.26 ppm, CD_2Cl_2 δ 5.32 ppm, or CD_3CN δ 1.94 ppm). ^{1}H NMR spectra are reported as follows: chemical shift (δ, ppm), multiplicity (s = singlet, d = doublet, t = triplet, q = quartet, dd = doublet of doublets, dt = doublet of triplets, dq = doublet of quartets, td = triplet of doublets, pd = pentet of doublets, ddd = doublet of doublet of doublets, ddt = doublet of doublet of triplets, tdd = triplet of doublet of doublets, m = multiplet), coupling constant(s) in Hz, and integration. Significant peaks are reported within the overlapping ~2.10–0.60 ppm region of the ^{1}H NMR spectra. ^{13}C NMR spectra are reported in chemical shifts downfield from TMS using the respective residual solvent peak as internal standard ($CDCl_3$ δ 77.16 ppm, CD_2Cl_2 δ 53.84 ppm, or CD_3CN δ 1.32 ppm and 118.26 ppm). ^{19}F NMR spectra are reported in chemical shifts upfield from TMS using $CFCl_3$ as internal standard. Line broadening parameters were 0.5 or 1.0 Hz, while the error of chemical shift value was 0.1 ppm.

Infrared spectra in the mid infrared region were recorded for KBr tablets on an IFS 113v FT-IR spectrophotometer (Bruker) equipped with a DTGS detector, and are reported as follows: wavenumbers (cm^{-1}), description (w = weak, m = medium, s = strong, br = broad). The spectra were taken at a resolution 2 cm^{-1}, NSS = 64. The Happ-Genzel apodization function was used.

Electrospray ionization (ESI) mass spectra were recorded on a Waters/Micromass ZQ mass spectrometer (Waters Alliance) equipped with a Harvard syringe pump. Samples were prepared in dry acetonitrile, and were infused into the ESI source using a Harvard pump at a flow rate of 20 mL min^{-1}. The ESI source potentials were: capillary 3 kV, lens 0.5 kV, and extractor 4 V. Standard ESI mass spectra were recorded at the cone voltages of 10 and 30 V. The source temperature was 120 °C and the desolvation temperature was 300 °C. Nitrogen was used as the nebulizing and desolvation gas at flow-rates of 100 dm^3 h^{-1}. Mass spectra were acquired in the positive ion detection mode with unit mass resolution at a step of 1 m/z unit. The mass range for ESI experiments was from m/z = 300 to m/z = 1100, or m/z = 300 to m/z = 1300. High-resolution mass spectra (HRMS) were recorded on a QTOF mass spectrometer (Impact HD, Bruker Daltonics).

Spectroscopic analysis of newly synthesized compounds

List of spectra

Figure S1. The ^{13}C NMR spectrum of precursor **2** in chloroform-d – S4 –
Figure S2. The ^{1}H NMR spectrum of precursor **2** in chloroform-d – S4 –
Figure S3. The ^{19}F NMR spectrum of precursor **2** in chloroform-d – S4 –
Figure S4. The ^{13}C NMR spectrum of precursor **3** in chloroform-d – S5 –
Figure S5. The ^{1}H NMR spectrum of precursor **3** in chloroform-d – S5 –
Figure S6. The ^{19}F NMR spectrum of precursor **3** in chloroform-d – S5 –
Figure S7. The ^{13}C NMR spectrum of bioconjugate **6** in chloroform-d – S6 –
Figure S8. The ^{1}H NMR spectrum of bioconjugate **6** in chloroform-d – S6 –
Figure S9. The ^{13}C NMR spectrum of bioconjugate **7** in dichloromethane-d_2 – S7 –
Figure S10. The ^{1}H NMR spectrum of bioconjugate **7** in dichloromethane-d_2 – S7 –
Figure S11. The ^{13}C NMR spectrum of bioconjugate **8** in chloroform-d – S8 –
Figure S12. The ^{1}H NMR spectrum of bioconjugate **8** in chloroform-d – S8 –
Figure S13. The ^{19}F NMR spectrum of bioconjugate **8** in chloroform-d – S8 –
Figure S14. The ^{13}C NMR spectrum of bioconjugate **9** in chloroform-d – S9 –
Figure S15. The ^{1}H NMR spectrum of bioconjugate **9** in chloroform-d – S9 –
Figure S16. The ^{19}F NMR spectrum of bioconjugate **9** in chloroform-d – S9 –
Figure S17. The ^{13}C NMR spectrum of bioconjugate **10** in chloroform-d – S10 –
Figure S18. The ^{1}H NMR spectrum of bioconjugate **10** in chloroform-d – S10 –
Figure S19. The ^{19}F NMR spectrum of bioconjugate **10** in chloroform-d – S10 –
Figure S20. The ^{13}C NMR spectrum of bioconjugate **11** in chloroform-d – S11 –
Figure S21. The ^{1}H NMR spectrum of bioconjugate **11** in chloroform-d – S11 –
Figure S22. The ^{19}F NMR spectrum of bioconjugate **11** in chloroform-d – S11 –
Figure S23. The ^{13}C NMR spectrum of bioconjugate **12** in chloroform-d – S12 –
Figure S24. The ^{1}H NMR spectrum of bioconjugate **12** in chloroform-d – S12 –
Figure S25. The ^{19}F NMR spectrum of bioconjugate **12** in chloroform-d – S12 –
Figure S26. The ^{13}C NMR spectrum of bioconjugate **13** in acetonitrile-d_3 – S13 –
Figure S27. The ^{1}H NMR spectrum of bioconjugate **13** in acetonitrile-d_3 – S13 –
Figure S28. The ^{31}P NMR spectrum of bioconjugate **13** in acetonitrile-d_3 – S13 –
Figure S29. The ^{13}C NMR spectrum of bioconjugate **14** in chloroform-d – S14 –
Figure S30. The ^{1}H NMR spectrum of bioconjugate **14** in chloroform-d – S14 –

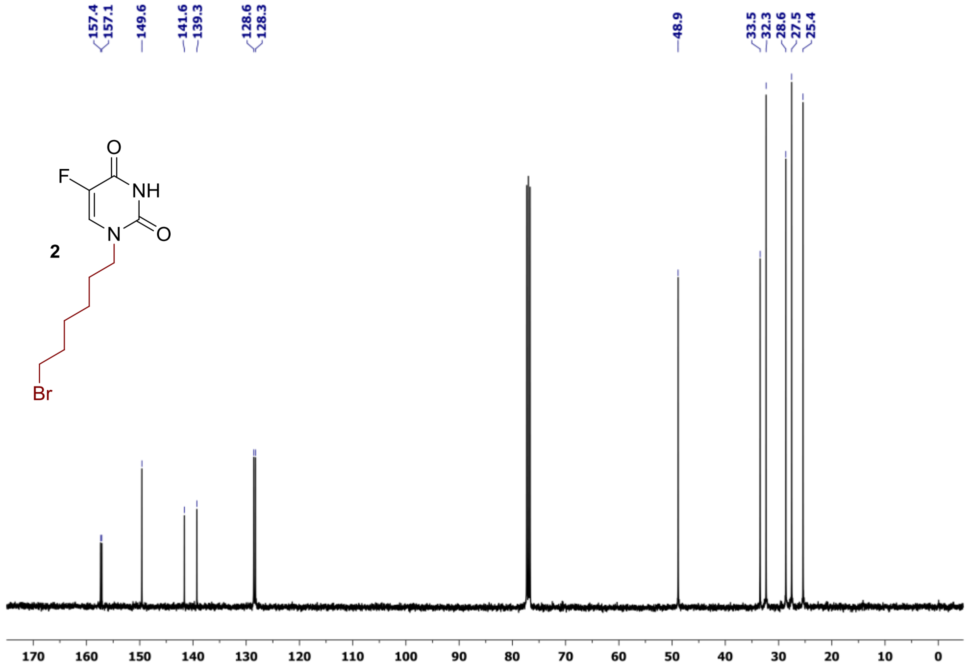

Figure S1. The ^{13}C NMR spectrum of precursor **2** in chloroform-d.

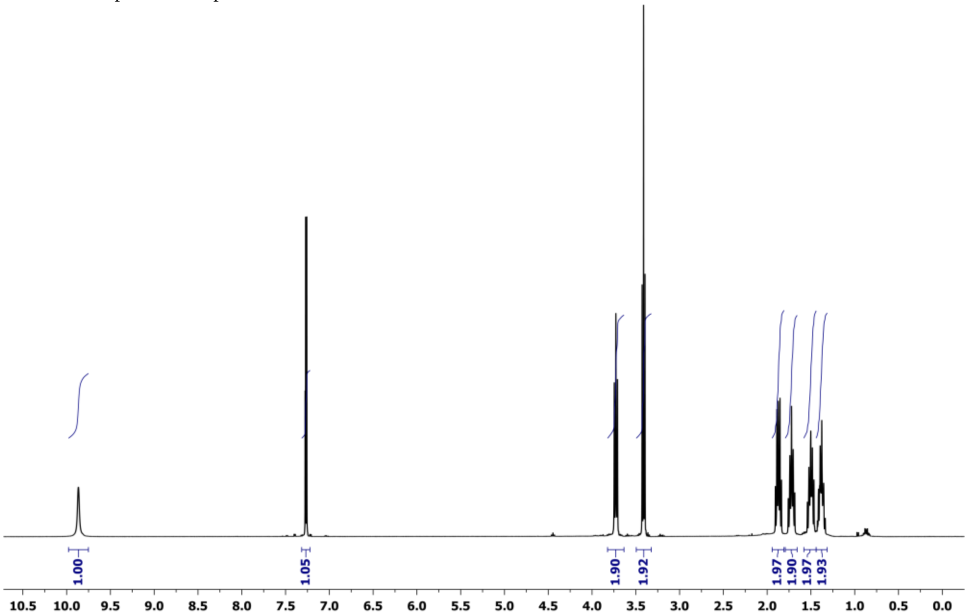

Figure S2. The ^{1}H NMR spectrum of precursor **2** in chloroform-d.

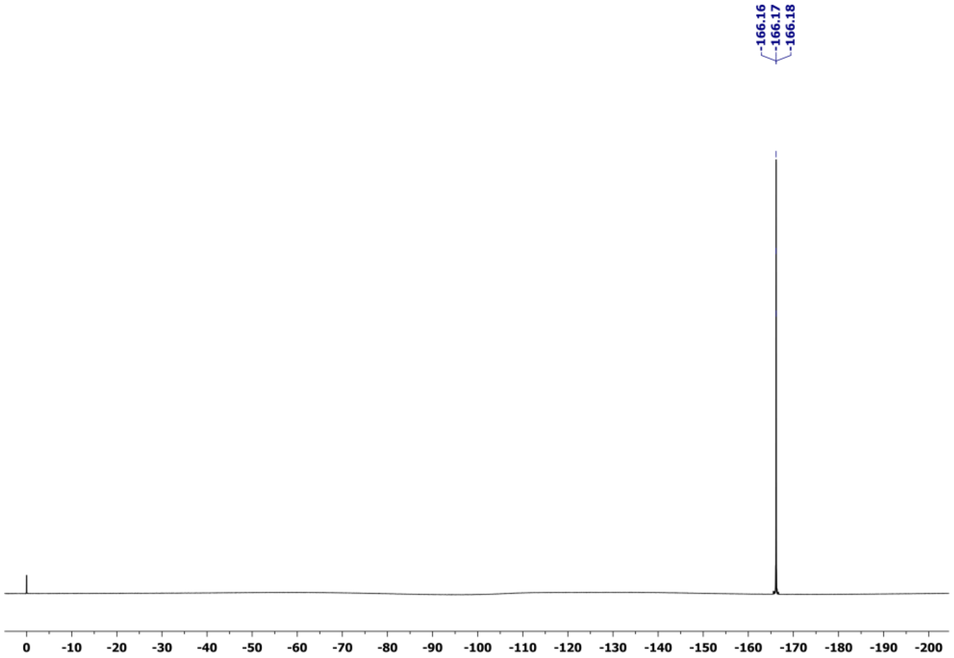

Figure S3. The ^{19}F NMR spectrum of precursor **2** in chloroform-d.

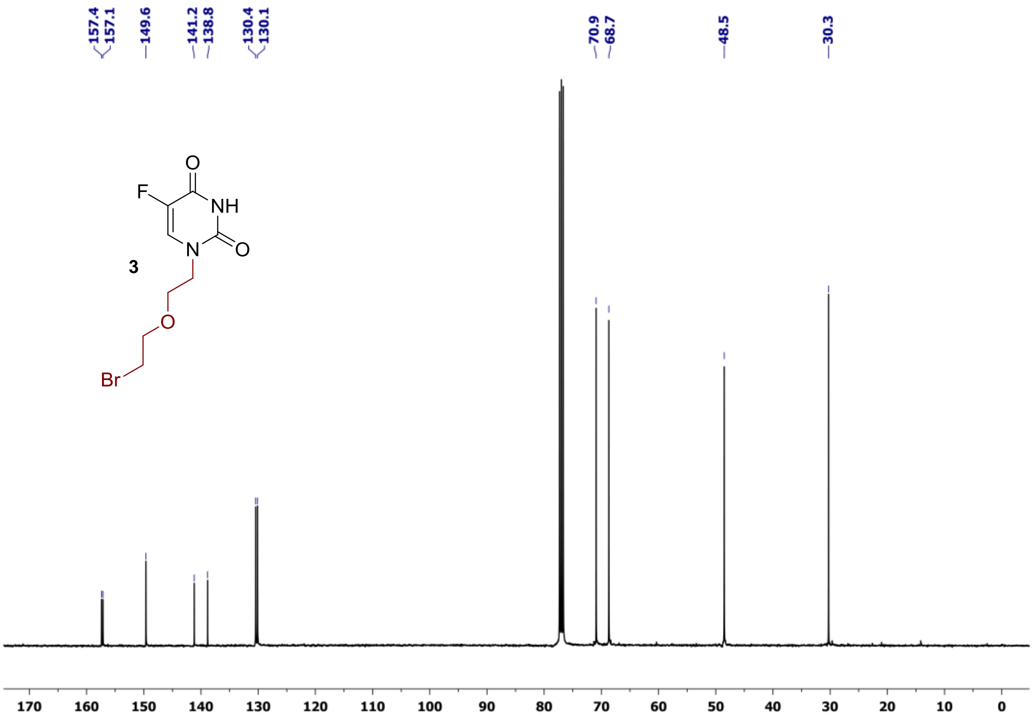

Figure S4. The ^{13}C NMR spectrum of precursor **3** in chloroform-d.

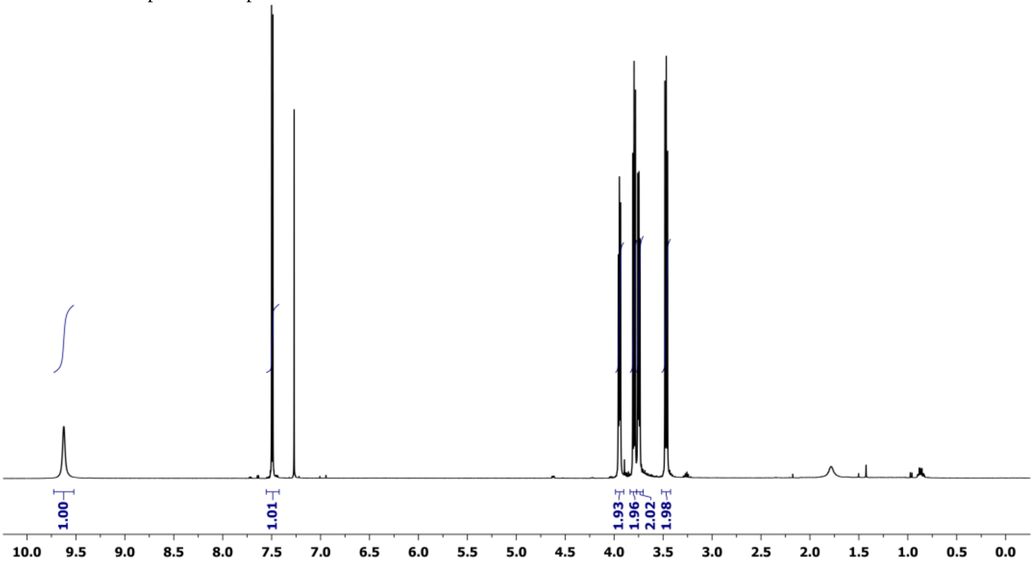

Figure S5. The ^{1}H NMR spectrum of precursor **3** in chloroform-d.

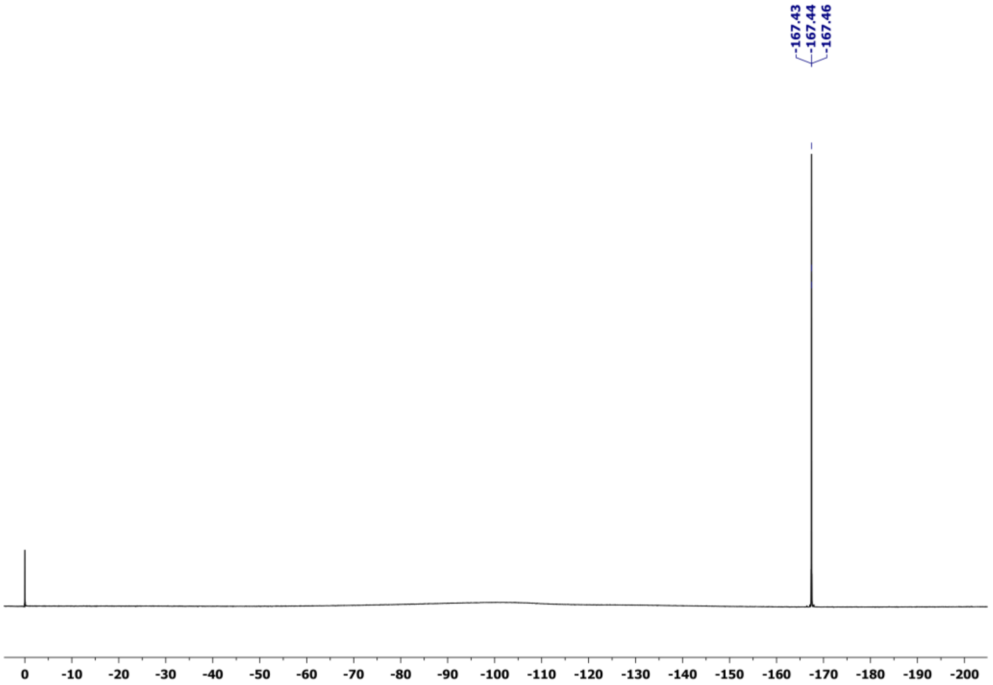

Figure S6. The ^{19}F NMR spectrum of precursor **3** in chloroform-d.

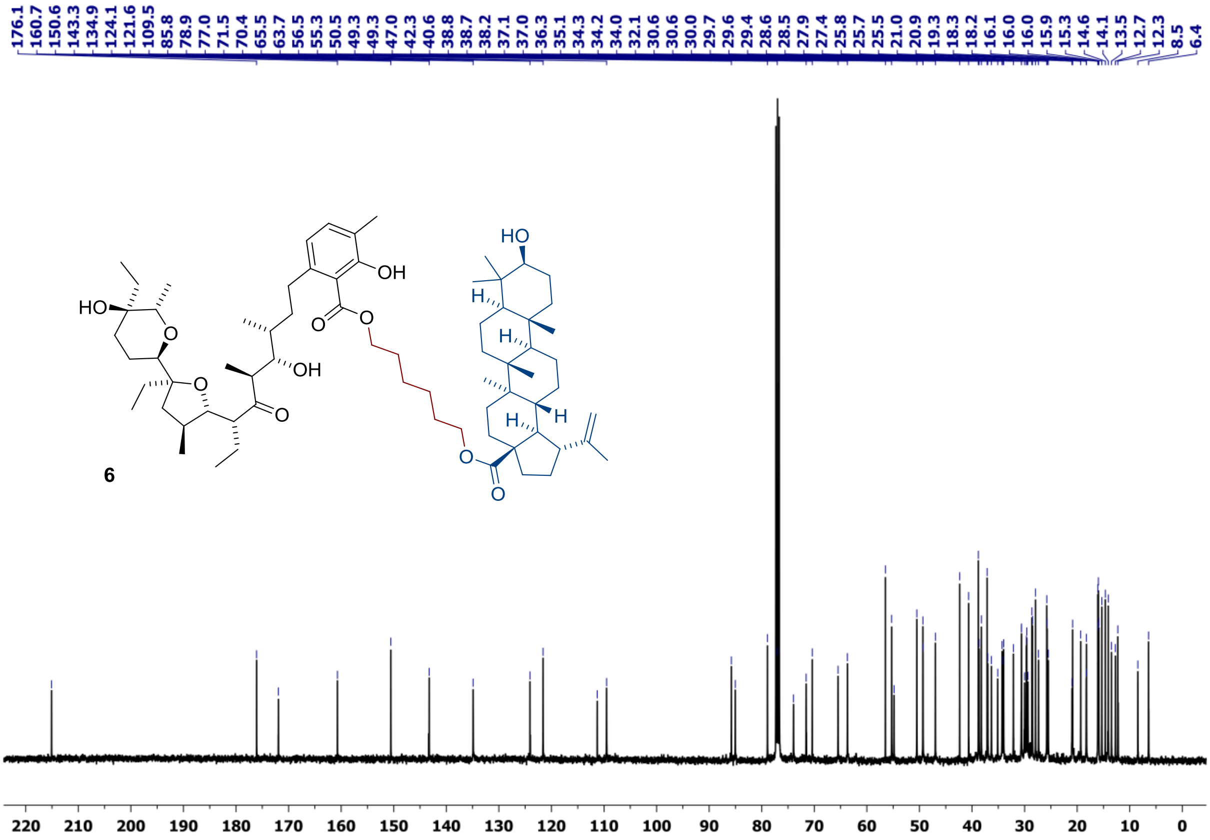

Figure S7. The ^{13}C NMR spectrum of bioconjugate **6** in chloroform-d.

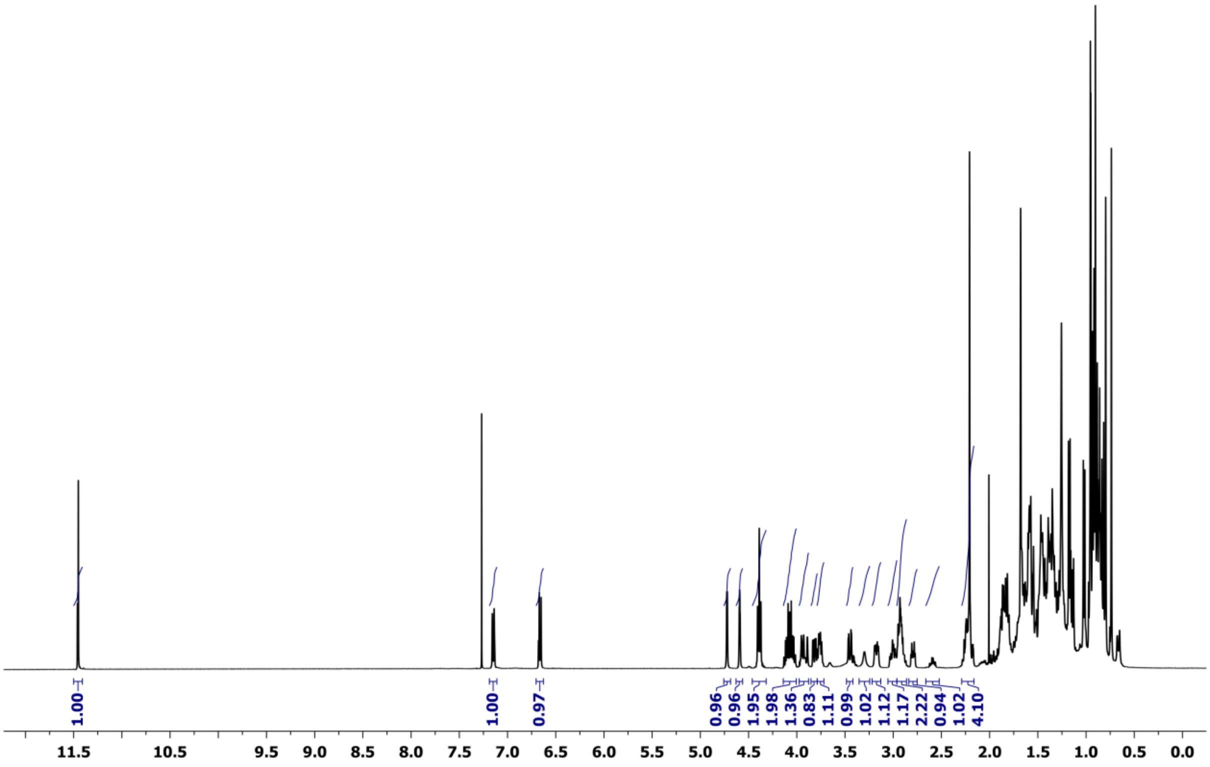

Figure S8. The ^1H NMR spectrum of bioconjugate **6** in chloroform-d.

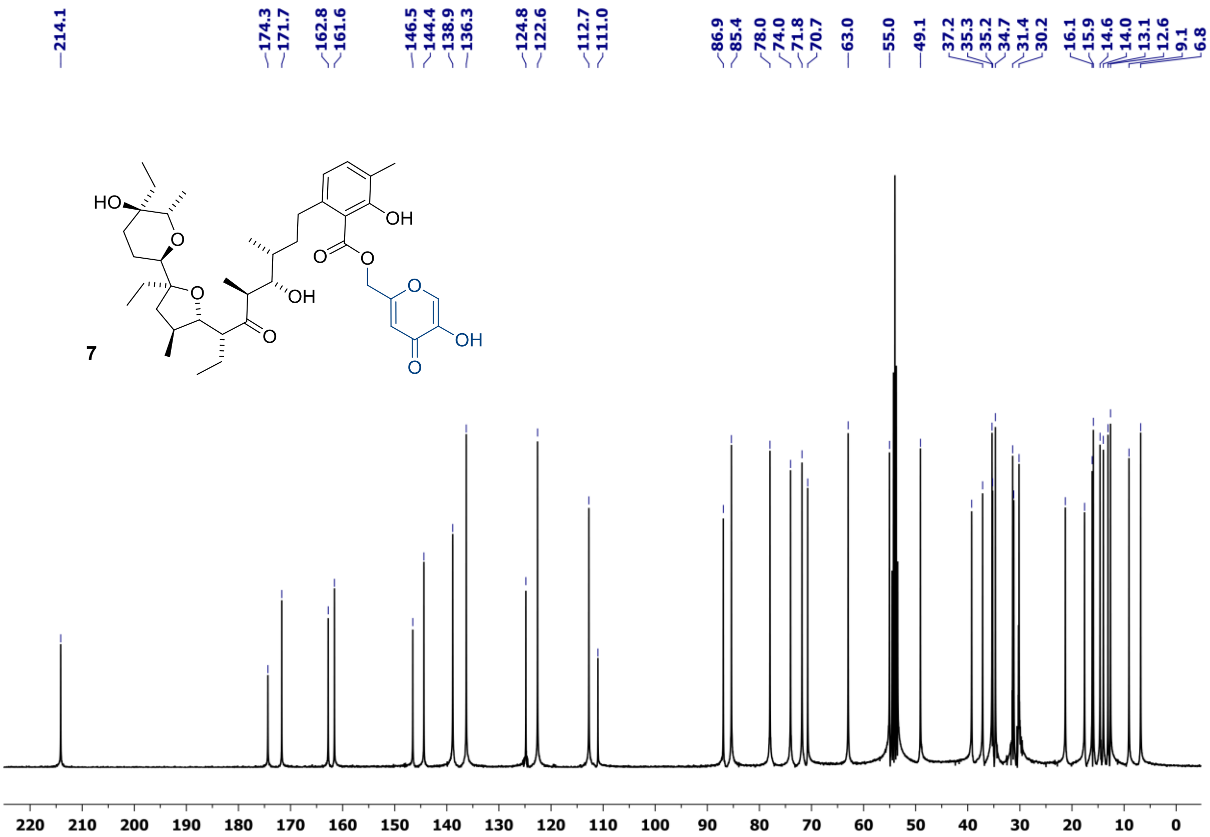

Figure S9. The ^{13}C NMR spectrum of bioconjugate **7** in dichloromethane-d_2.

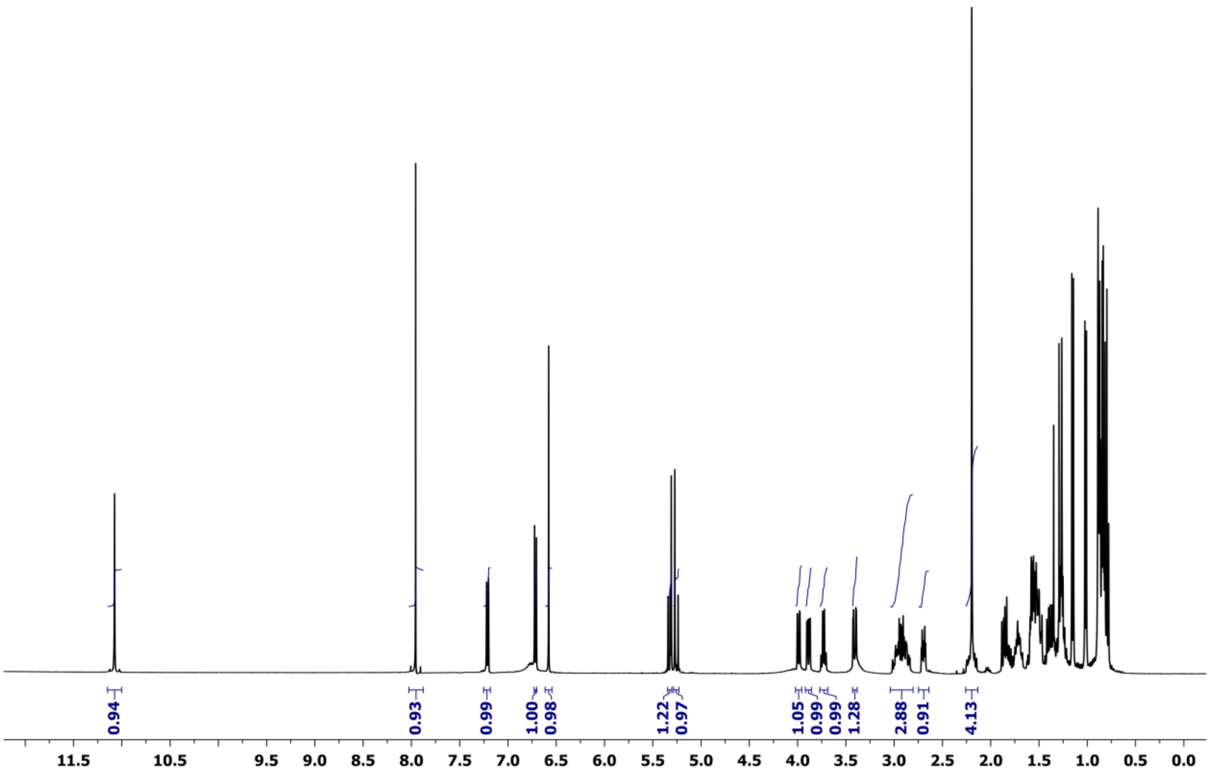

Figure S10. The ^{1}H NMR spectrum of bioconjugate **7** in dichloromethane-d_2.

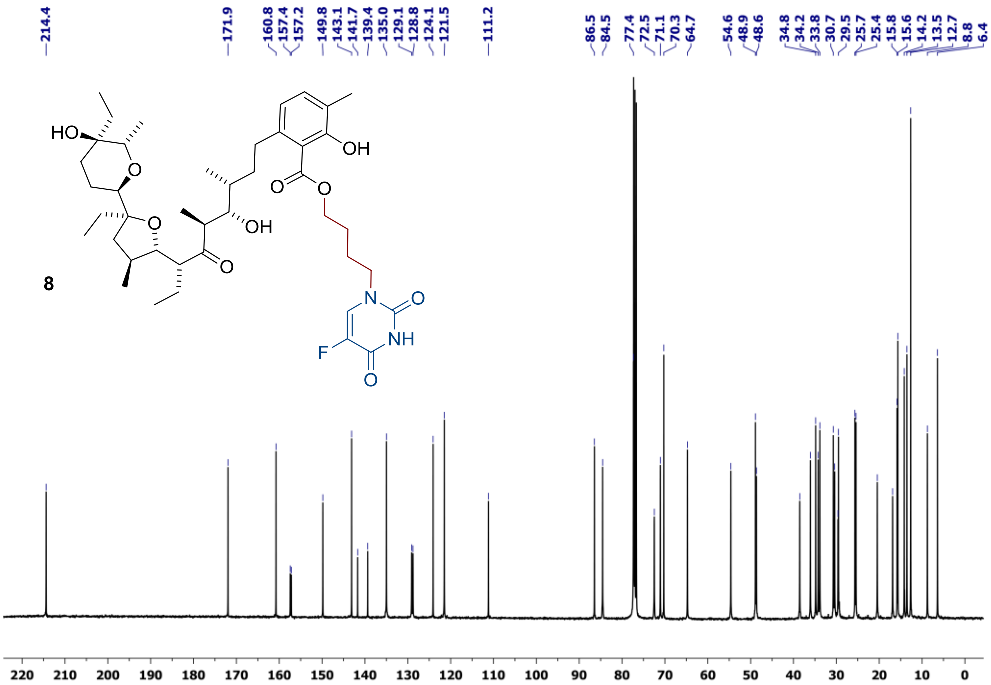

Figure S11. The ^{13}C NMR spectrum of bioconjugate **8** in chloroform-d.

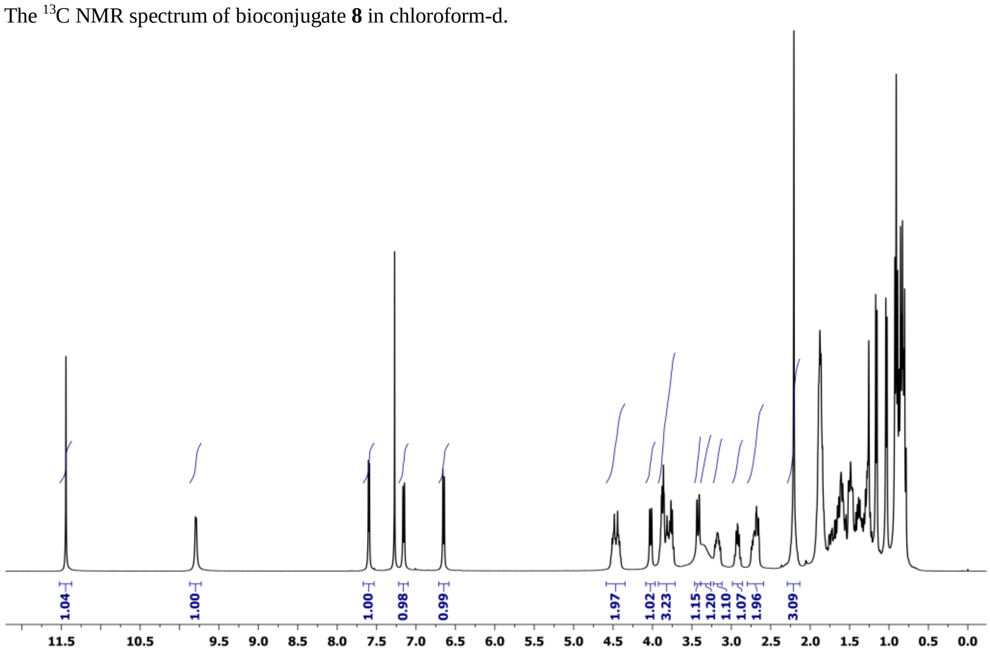

Figure S12. The ^{1}H NMR spectrum of bioconjugate **8** in chloroform-d.

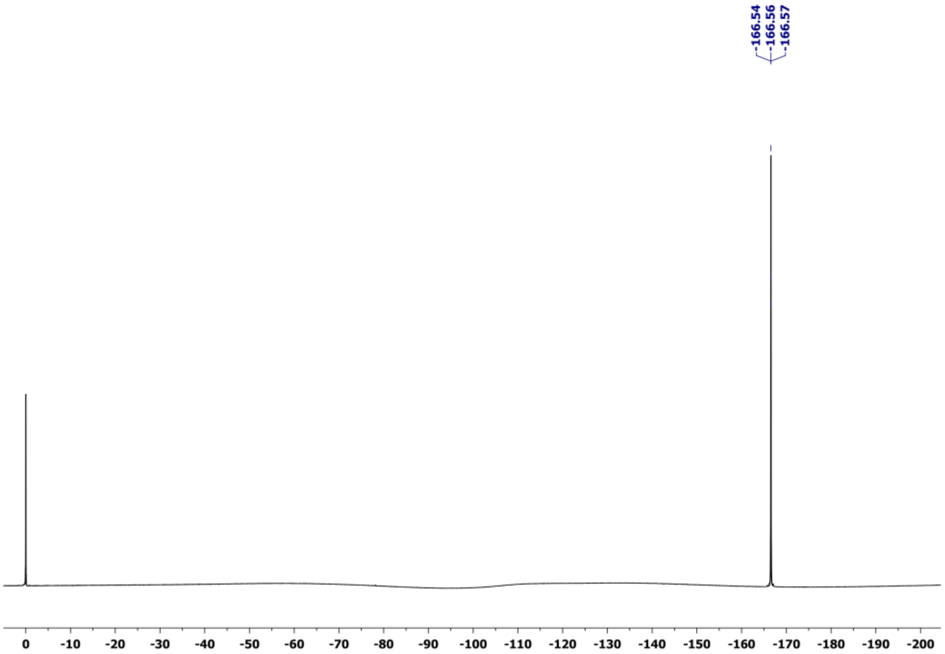

Figure S13. The ^{19}F NMR spectrum of bioconjugate **8** in chloroform-d.

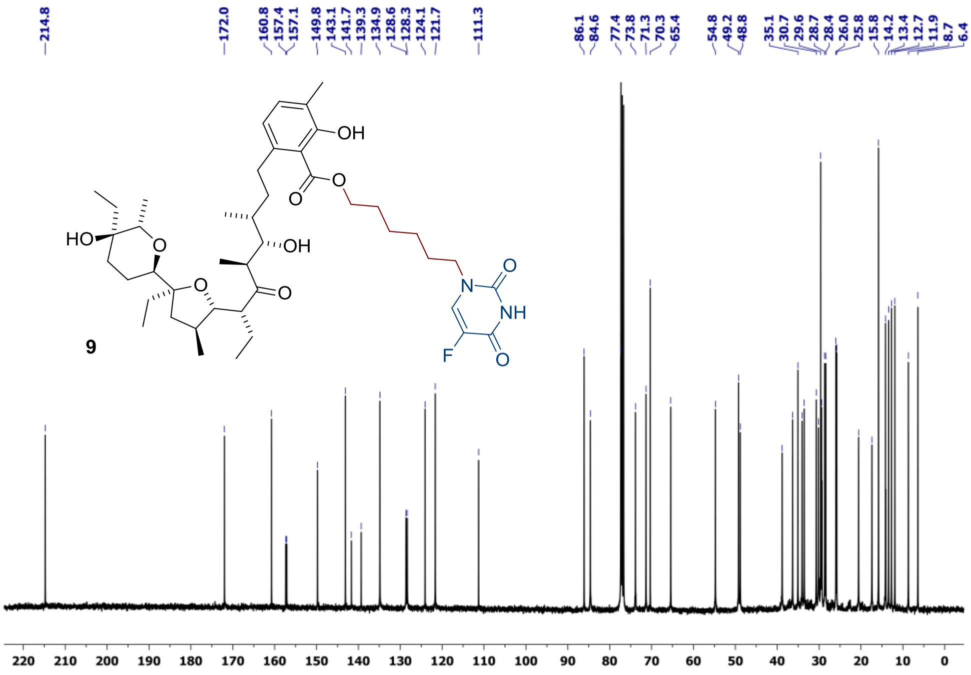

Figure S14. The ^{13}C NMR spectrum of bioconjugate **9** in chloroform-d.

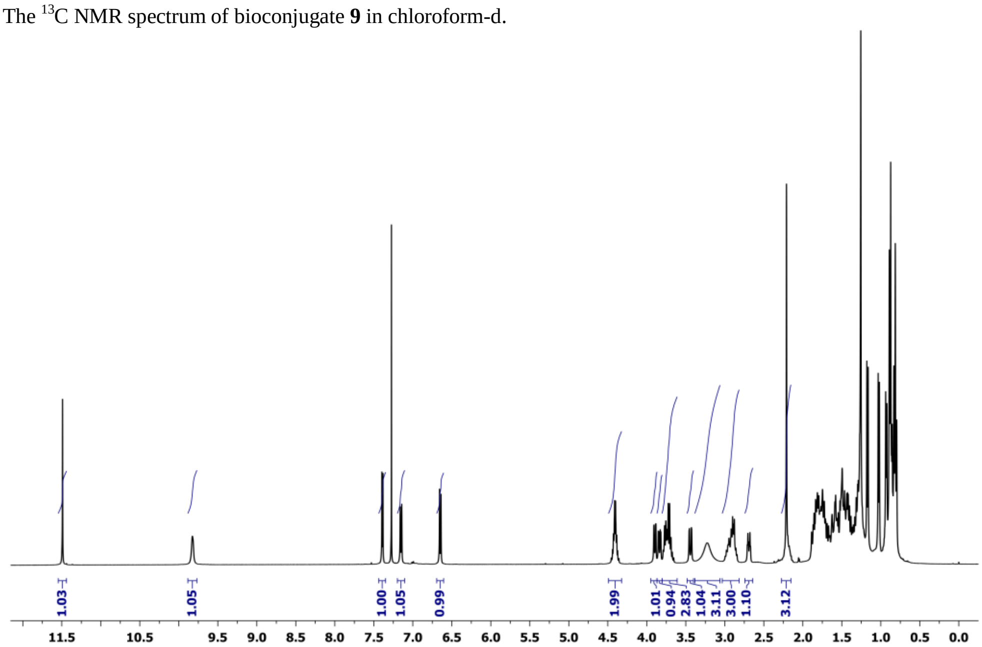

Figure S15. The ^{1}H NMR spectrum of bioconjugate **9** in chloroform-d.

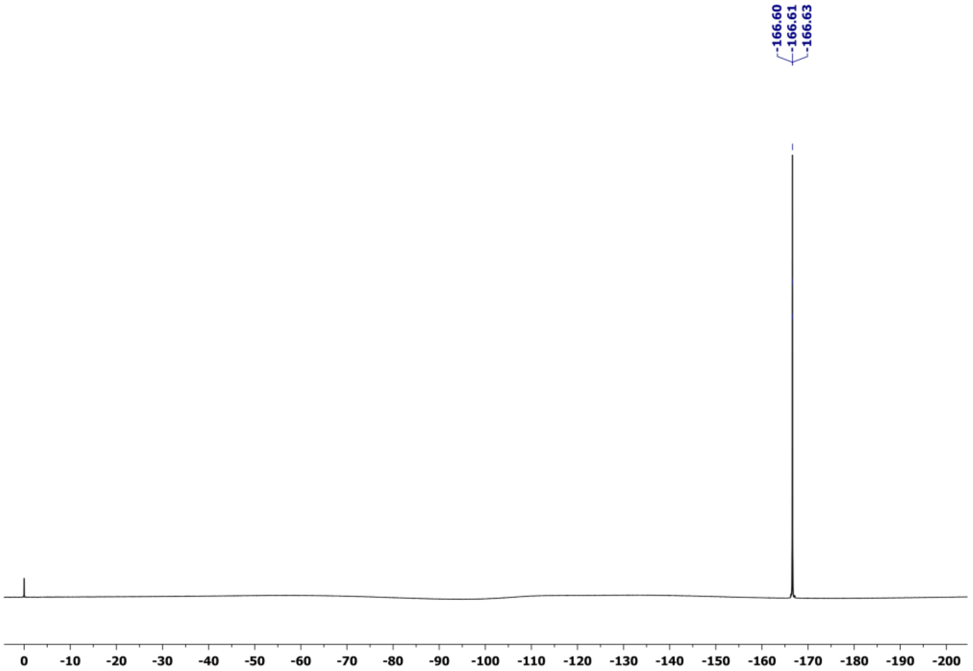

Figure S16. The ^{19}F NMR spectrum of bioconjugate **9** in chloroform-d.

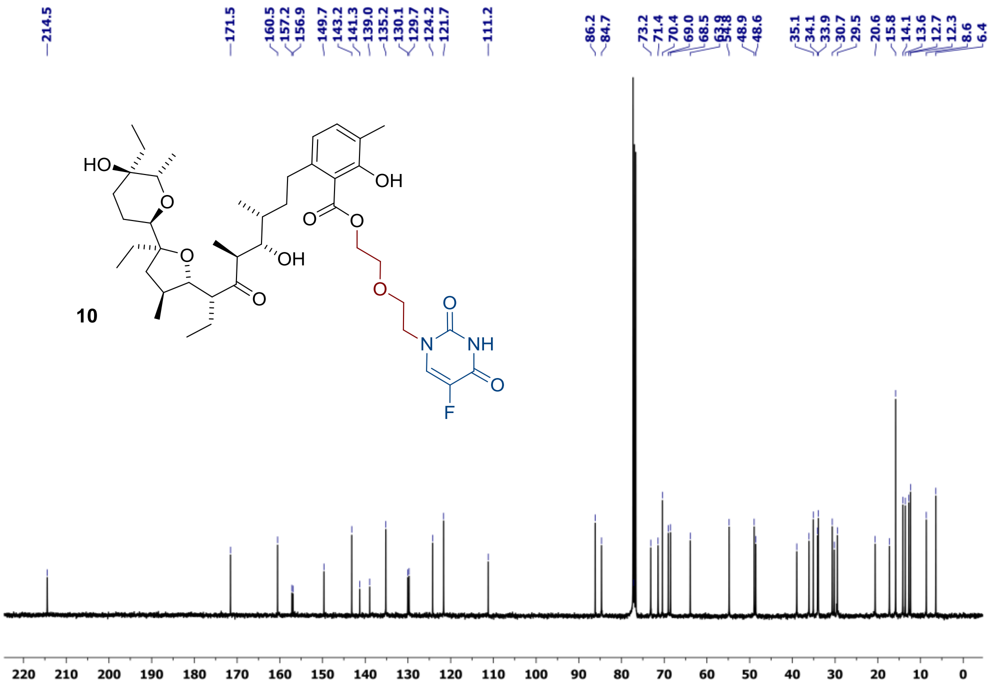

Figure S17. The ^{13}C NMR spectrum of bioconjugate **10** in chloroform-d.

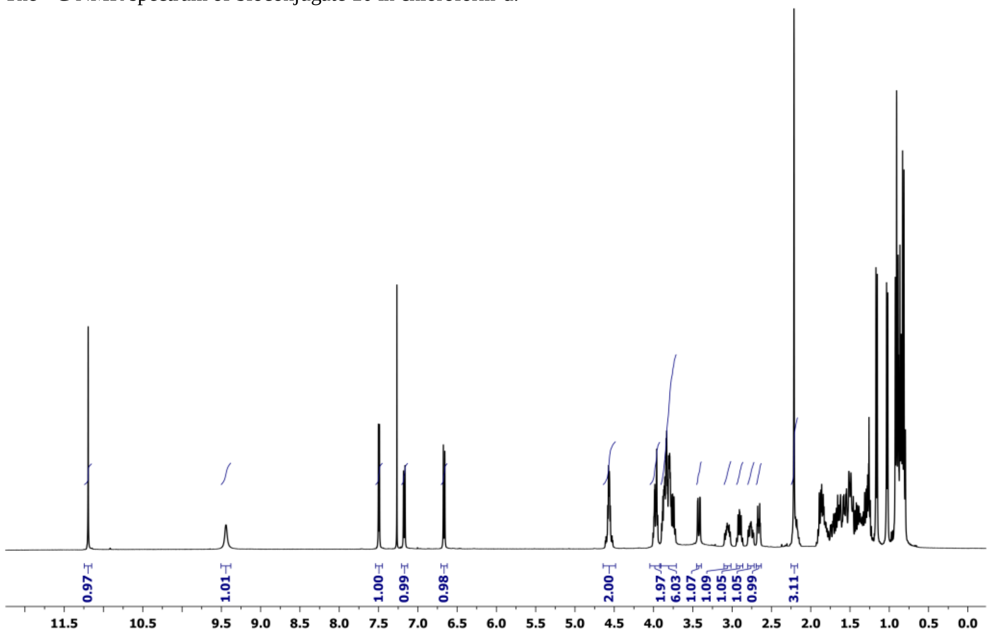

Figure S18. The ^{1}H NMR spectrum of bioconjugate **10** in chloroform-d.

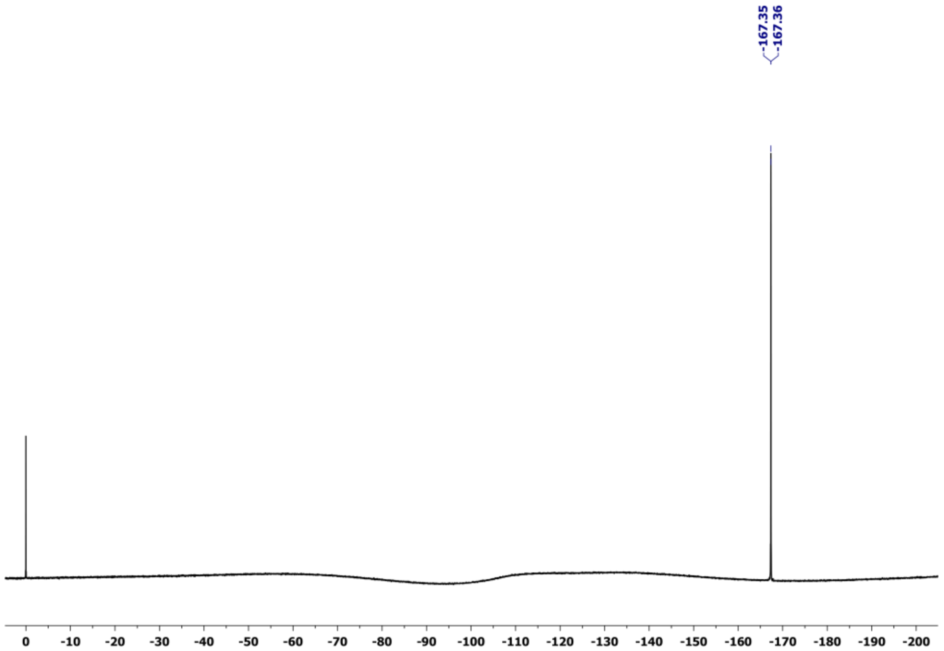

Figure S19. The ^{19}F NMR spectrum of bioconjugate **10** in chloroform-d.

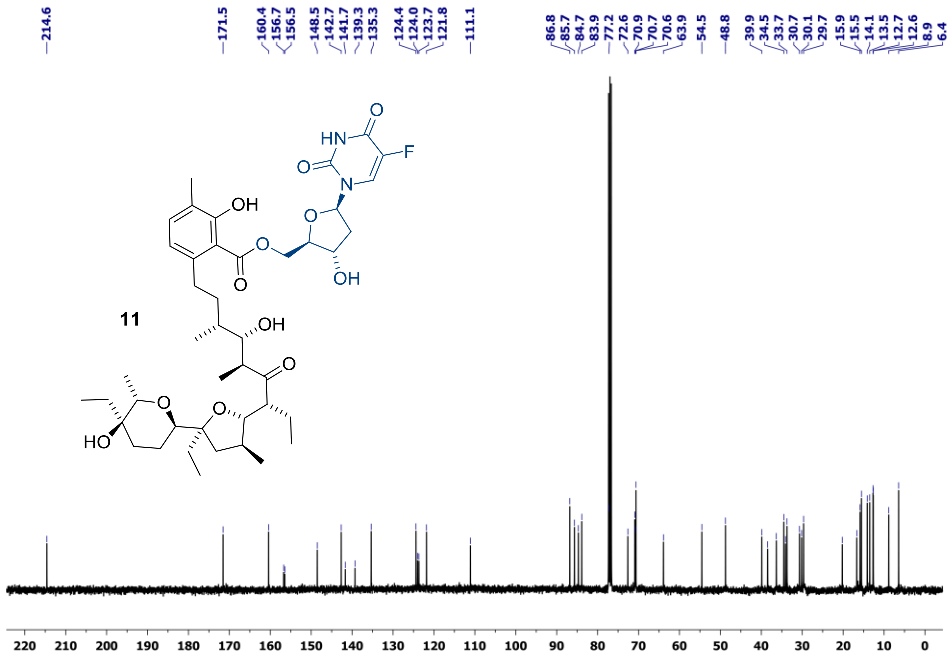

Figure S20. The ^{13}C NMR spectrum of bioconjugate **11** in chloroform-d.

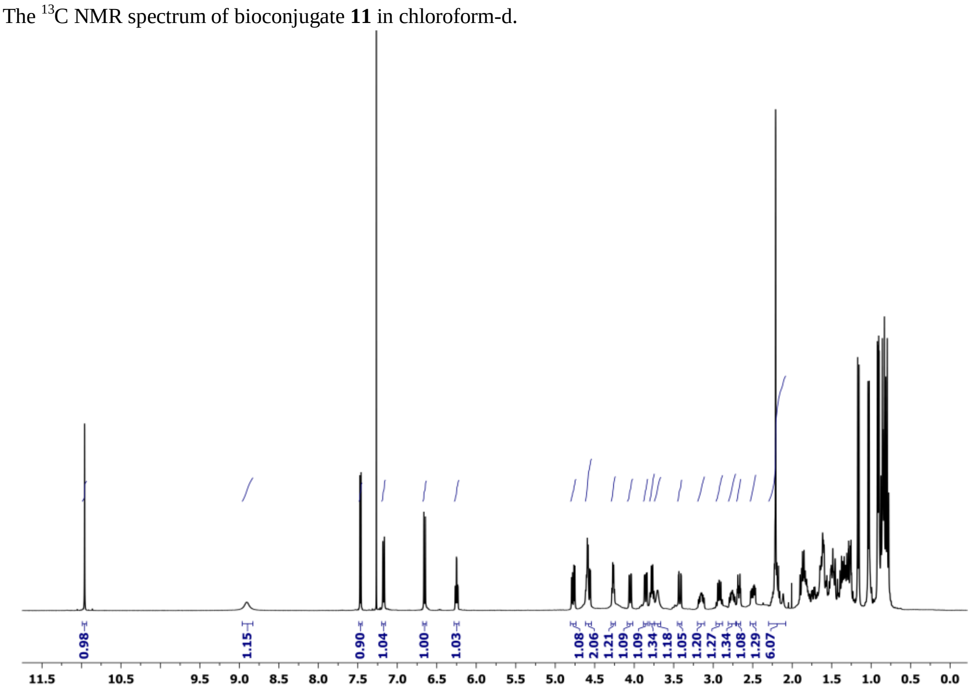

Figure S21. The ^{1}H NMR spectrum of bioconjugate **11** in chloroform-d.

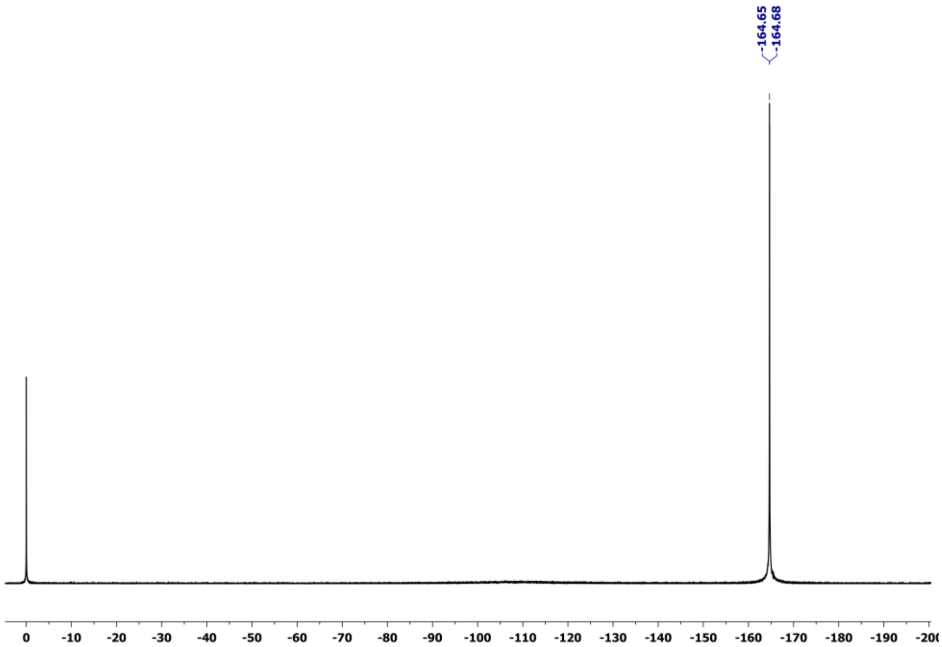

Figure S22. The ^{19}F NMR spectrum of bioconjugate **11** in chloroform-d.

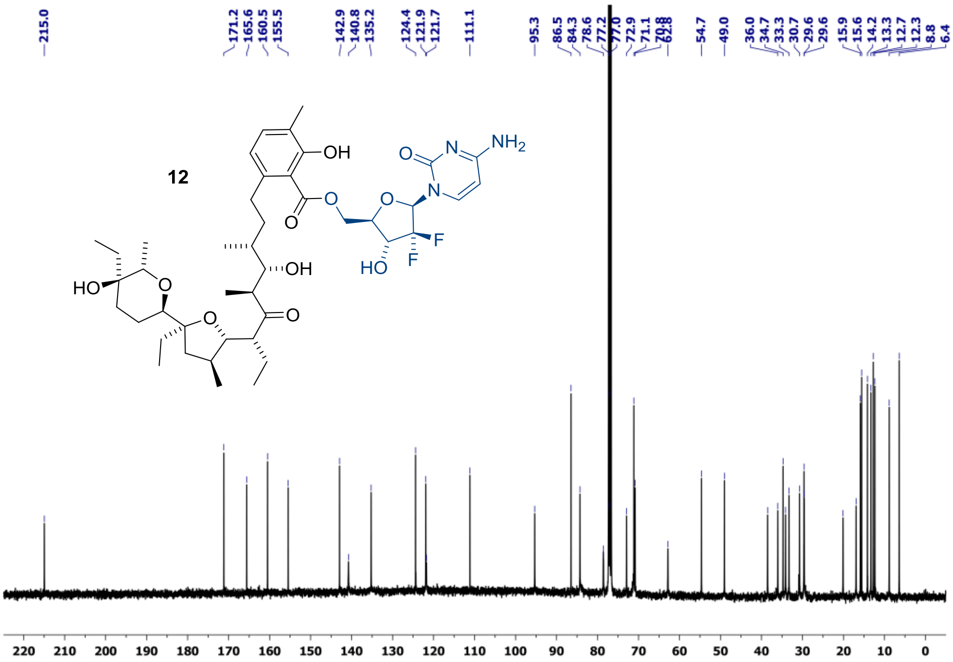

Figure S23. The ^{13}C NMR spectrum of bioconjugate **12** in chloroform-d.

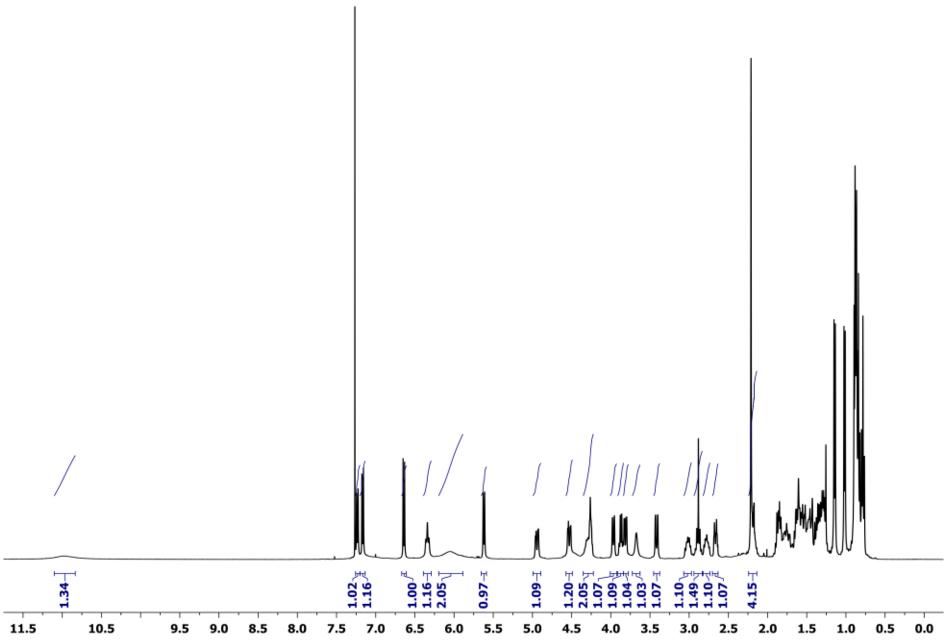

Figure S24. The ^{1}H NMR spectrum of bioconjugate **12** in chloroform-d.

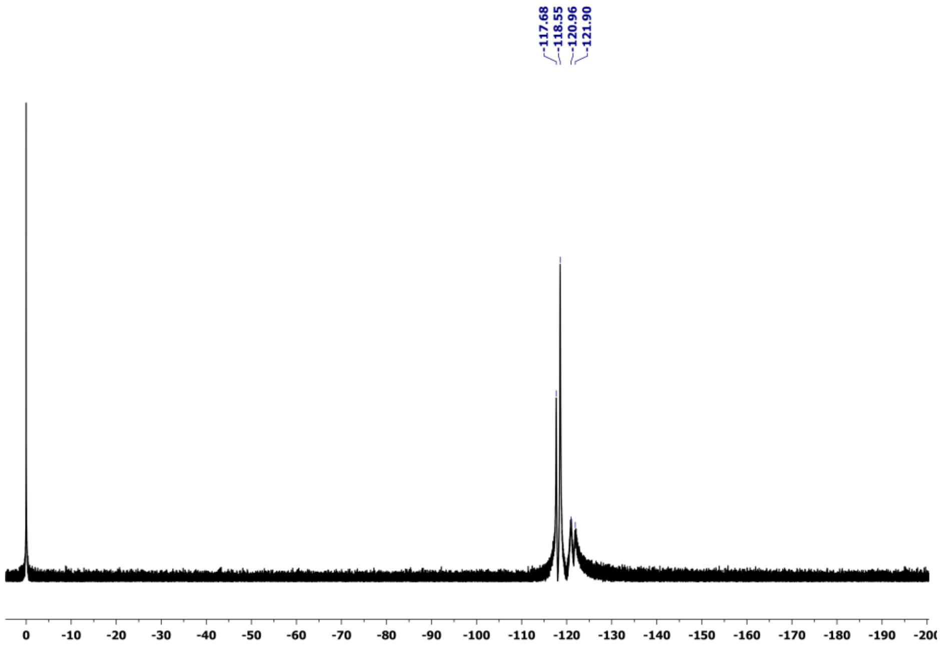

Figure S25. The ^{19}F NMR spectrum of bioconjugate **12** in chloroform-d.

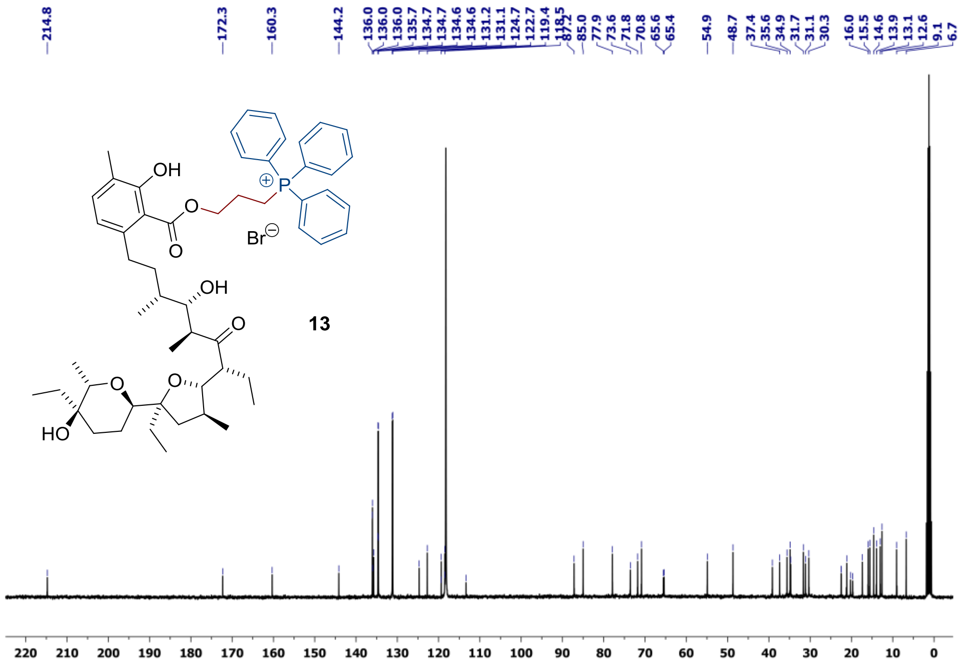

Figure S26. The ^{13}C NMR spectrum of bioconjugate **13** in acetonitrile-d$_3$.

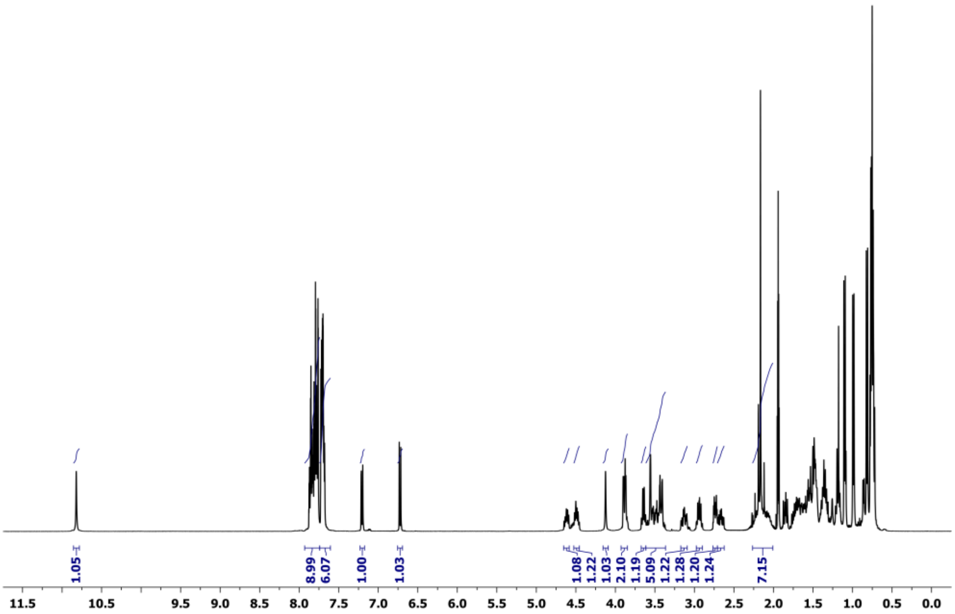

Figure S27. The ^{1}H NMR spectrum of bioconjugate **13** in acetonitrile-d$_3$.

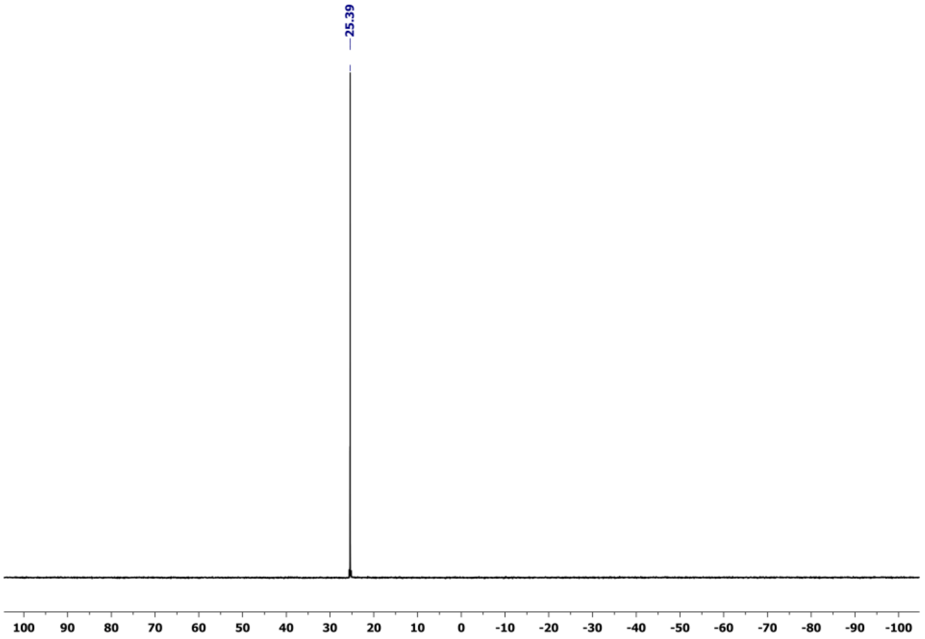

Figure S28. The ^{31}P NMR spectrum of bioconjugate **13** in acetonitrile-d$_3$.

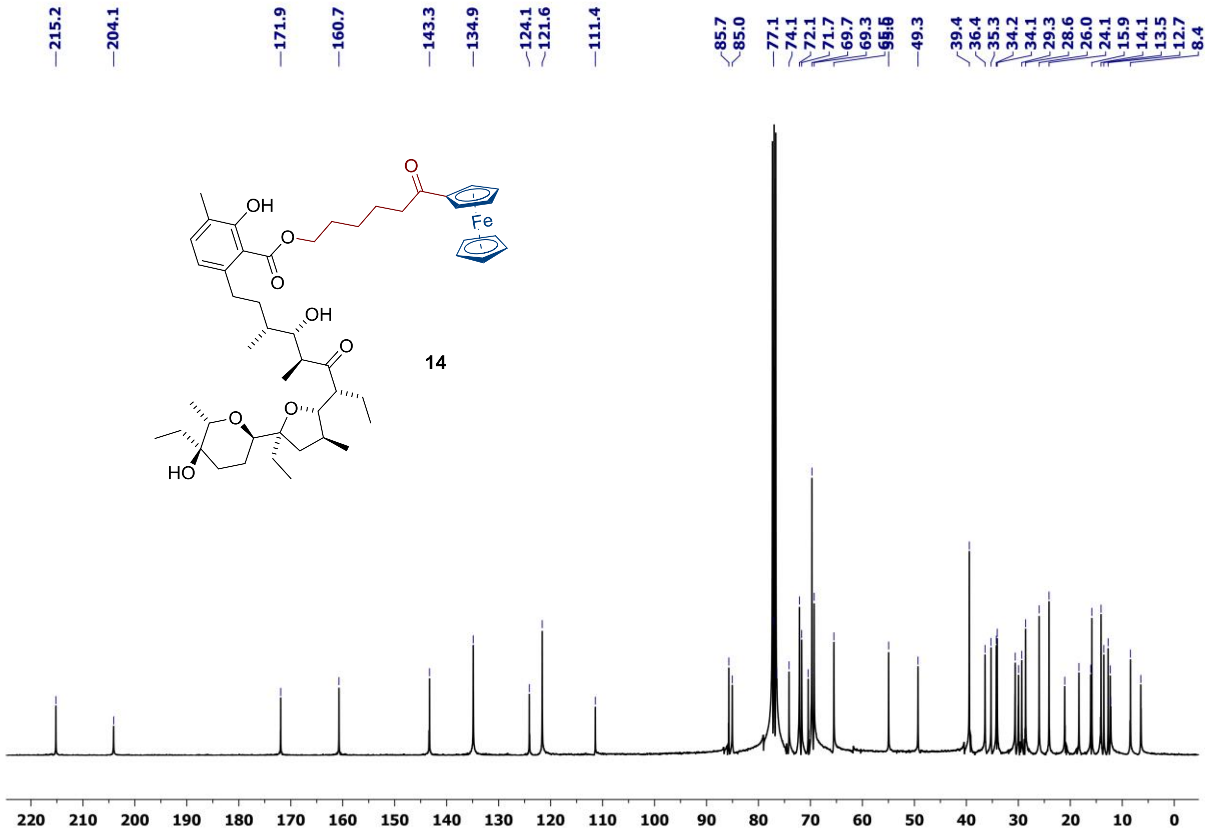

Figure S29. The ^{13}C NMR spectrum of bioconjugate **14** in chloroform-d.

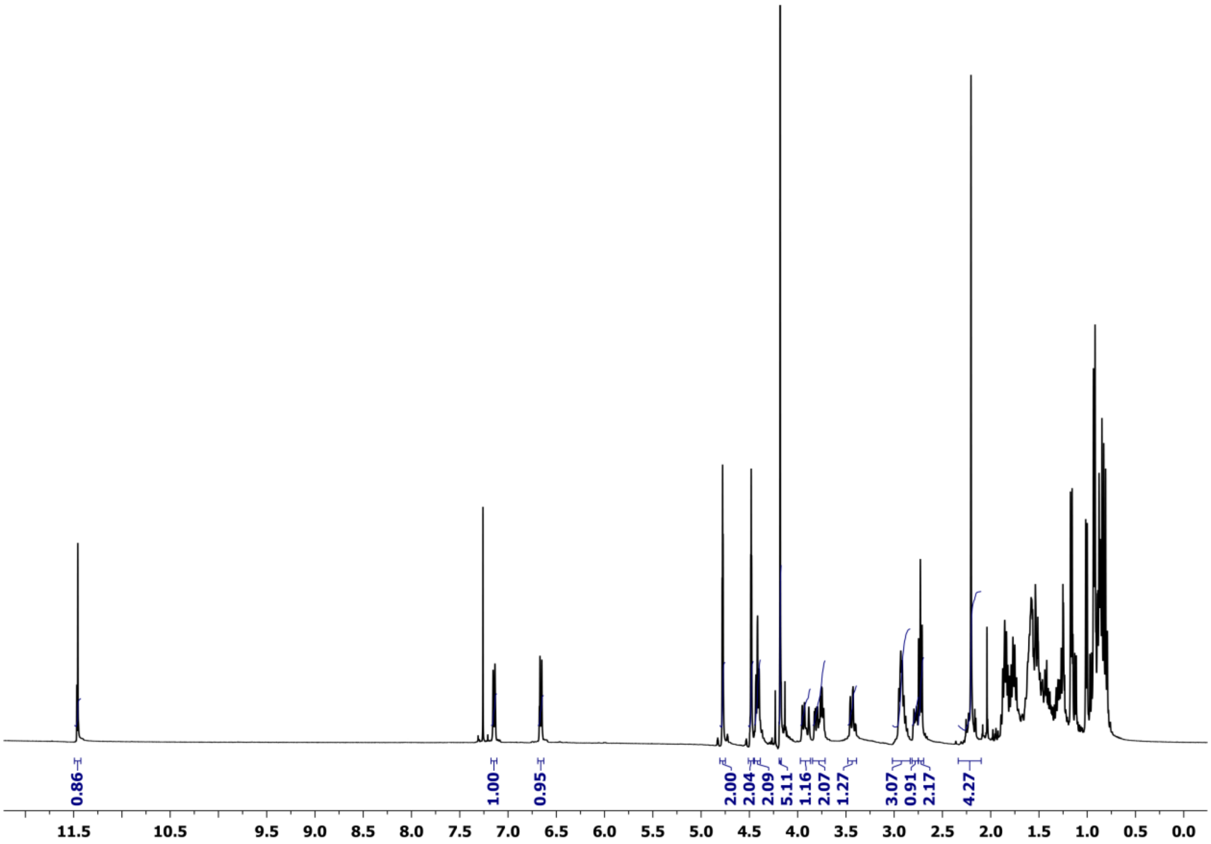

Figure S30. The ^{1}H NMR spectrum of bioconjugate **14** in chloroform-d.
